# Supplementary material for: Synthesis and Stereostructure-Activity Relationship of Novel Pyrethroids Possessing Two Asymmetric Centers on a Cyclopropane Ring
Source: Molecules. 2019 Mar 14;24(6):1023. doi: 10.3390/molecules24061023 (PMC6471473; doi:10.3390/molecules24061023)
Supplement: Supplementary file 1 [file molecules-24-01023-s001.zip › Molecules-Two asymmetric centers pyrethroid-HPLC.pdf]

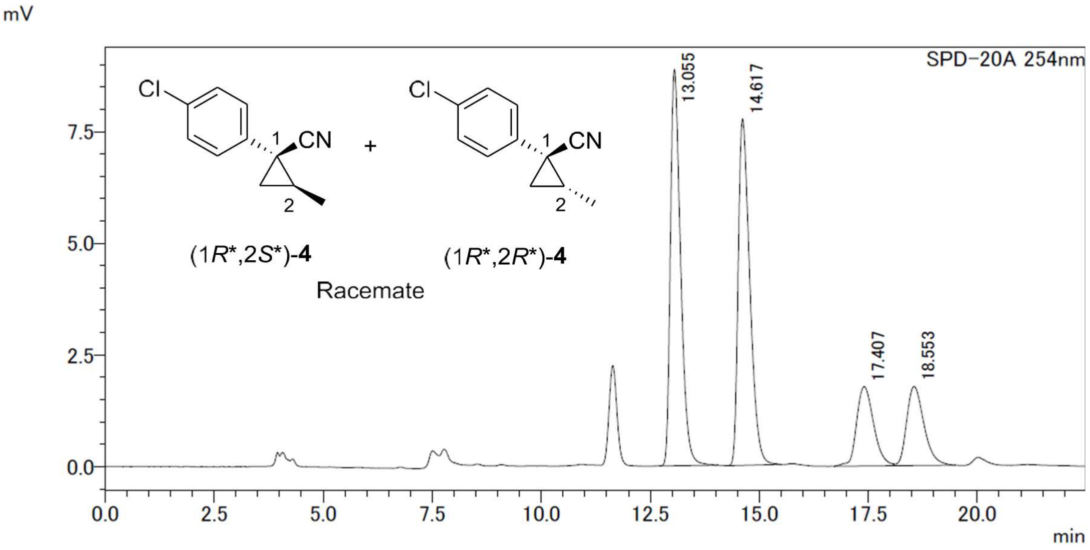

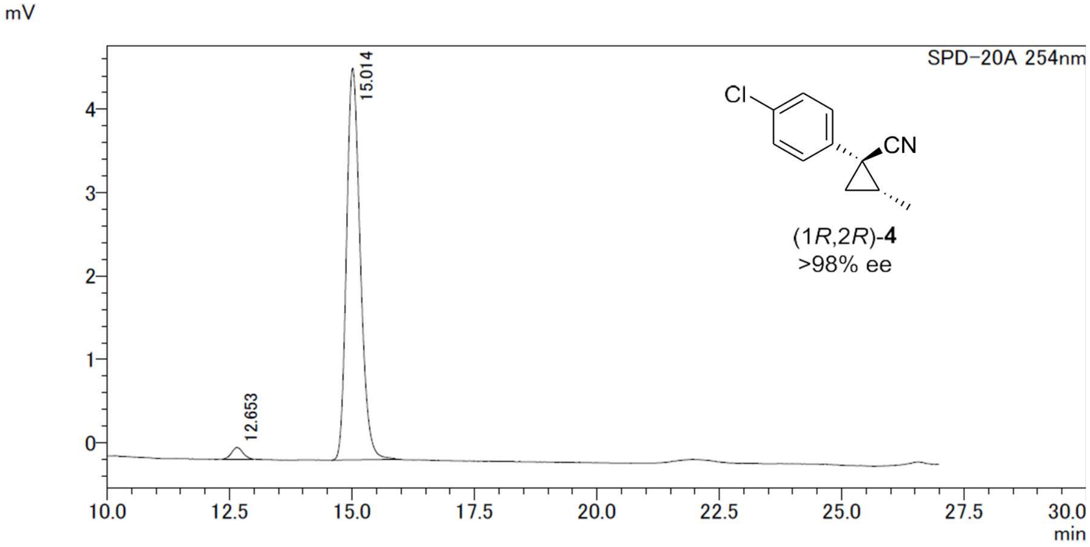

Peak	Retention Time	Area	Height	%	Unit	Mark	Compounds
1	3.910	1471	264	0.000			
2	3.983	1681	249	0.000		V	
3	12.635	2222	140	0.000			
4	15.014	91537	4696	0.000			
Total		96911	5349				

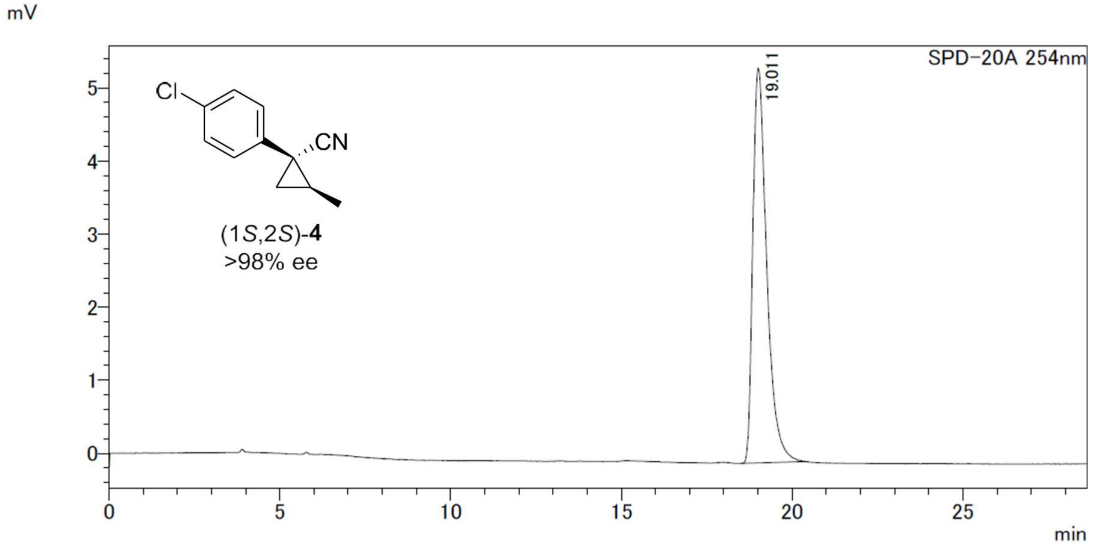

Peak	Retention Time	Area	Height	%	Unit	Mark	Compounds
1	19.011	152904	5403	0.000			
Total		152904	5403				

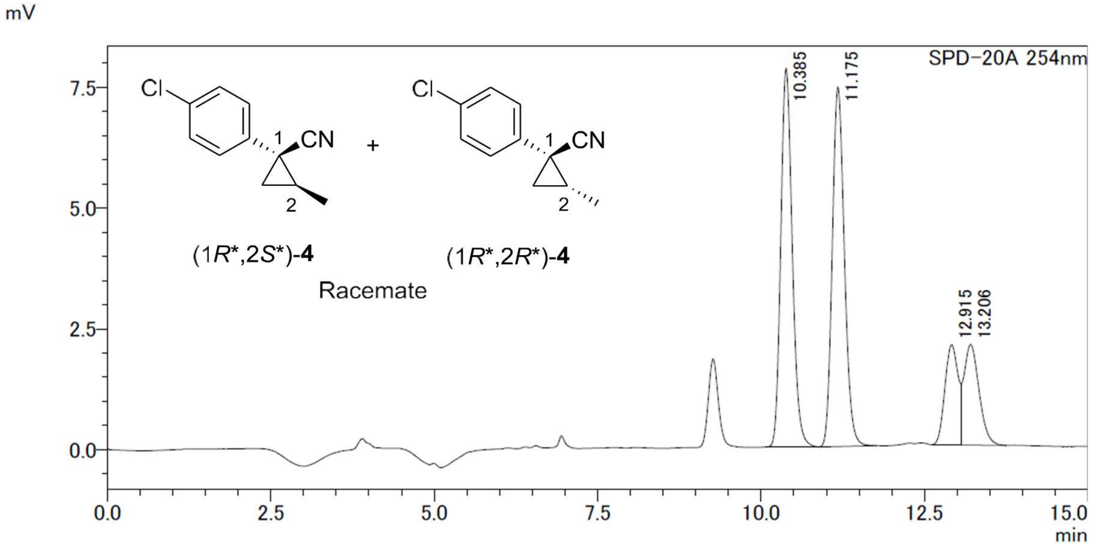

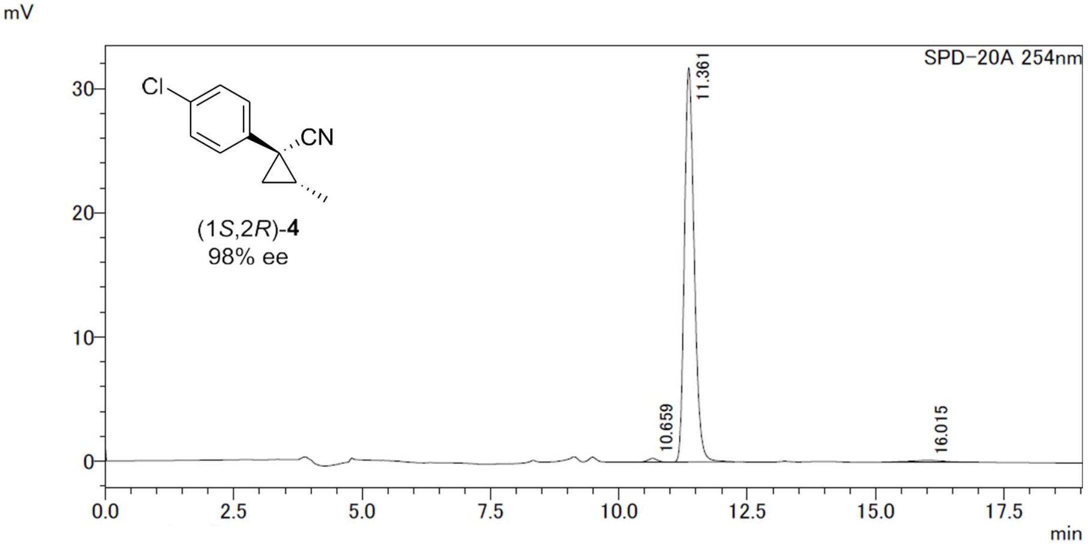

Peak	Retention Time	Area	Height	%	Unit	Mark	Compounds
1	10.659	3699	291	0.863		M	
2	11.361	420100	31570	97.986			
3	16.015	4938	127	1.152			
Total		428736	32168				

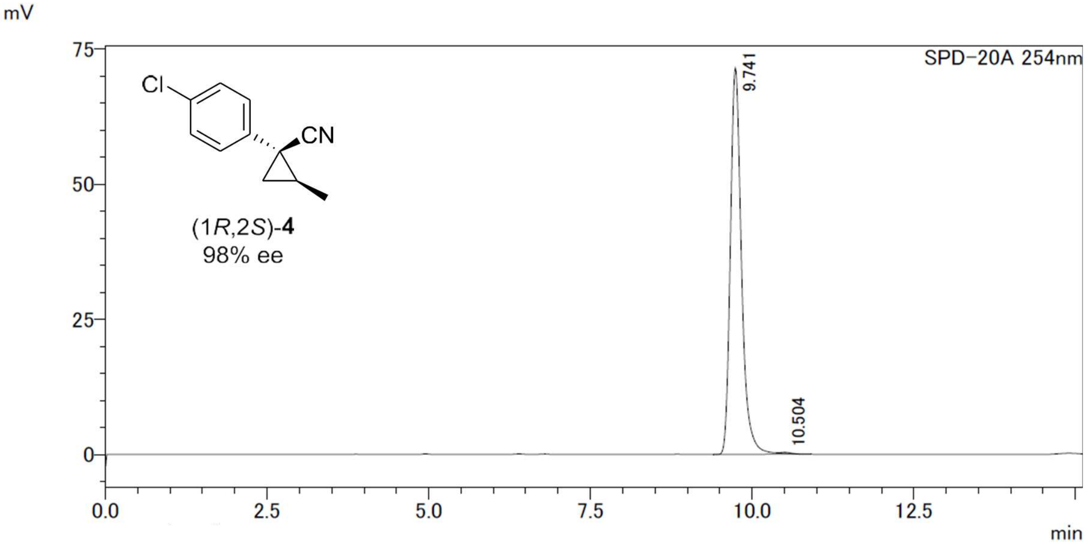

Peak	Retention Time	Area	Height	%	Unit	Mark	Compounds
1	9.741	852801	71434	99.809		S	
2	10.504	1634	177	0.191		T	
Total		854435	71608				
