# Supplementary material for: Synthesis and Stereostructure-Activity Relationship of Novel Pyrethroids Possessing Two Asymmetric Centers on a Cyclopropane Ring
Source: Molecules. 2019 Mar 14;24(6):1023. doi: 10.3390/molecules24061023 (PMC6471473; doi:10.3390/molecules24061023)
Supplement: Supplementary file 1 [file molecules-24-01023-s001.zip › Molecules-Two asymmetric certers pyrethroid-experimental-ESI.pdf]

Appendix A (Electronic Supporting Information)

Synthesis and stereostructure-activity relationship of novel pyrethroids possessing two asymmetric centers on a cyclopropane ring

Takashi Taniguchi, Yasuaki Taketomo, Mizuki Moriyama, Noritada Matsuo, Yoo Tanabe*

Department of Chemistry, School of Science and Technology, Kwansei Gakuin University, 2 -1 Gakuen, Sanda, Hyogo, 669-1337, Japan. Email: tanabe@kwansei.ac.jp.

1-(4-Chlorophenyl)cyclopropane-1-carbonitrile (11) [1]

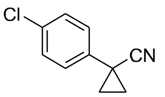

1,2-Dibromoethane (1.72 mL, 20 mmol) was added to a stirred suspension of 2-(4-chlorophenyl)acetonitrile (1.52 g, 10 mmol), tetrabuthyl ammonium bromide (TBAB) (161 mg, 0.05 eq.), and KOH (5.61 g, 100 mmol) in water (5 mL) at 70 – 75 °C under an Ar atmosphere, followed by being stirred at the same temperature for 24 h [2]. The mixture was cooled down to room temperature and reversely quenched with ice and 6 M-HCl aq., which was extracted twice with Et_2O. The combined organic phase was washed with water, brine, dried (Na_2SO_4) and concentrated. The obtained crude oil was purified by SiO_2-column chromatography (hexane/AcOEt = 15:1) to give the desired product **1** (1.42 g, 80%).

Pale yellow crystals: mp 55 – 56 °C [lit.[3] 42 – 45 °C]; ^{1}H NMR (500 MHz, $CDCl_3$): δ = 1.37-1.40 (m, 2H), 1.73-1.76 (m, 2H), 7.21-7.25 (m, 2H), 7.31-7.34 (m, 2H); ^{13}C NMR (125 MHz, $CDCl_3$): δ = 13.3, 18.2, 122.0, 127.1, 129.0, 133.5, 134.5.

(1-(4-Chlorophenyl)cyclopropyl)methanol (12) [4]

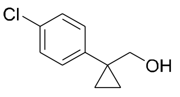

A mixture of nitrile **11** (1.42 g, 8.0 mmol) and NaOH (6.4 g, 160 mmol) in MeOH (40 mL) and water (26 mL) was stirred at 100 – 105 °C for 14 h under an Ar atmosphere. The mixture was cool down to room temperature and quenched with water, which was washed twice with Et_2O. 6 M-HCl aq. was added to adjust the separated water phase to pH = 1. Water phase was extracted twice with AcOEt, and the combined organic phase was washed with water, brine, dried (Na_2SO_4) and concentrated to give the desired carboxylic acid intermediate [4] (1.21g, 77%), which was sufficiently pure without any purification for the next step.

Pale yellow crystals: mp 153 – 155 °C [lit.[1] 154 – 156 °C]; ^{1}H NMR (500 MHz, $CDCl_3$): δ = 1.23-1.25 (m, 2H), 1.66-1.69 (m, 2H), 7.28 (s, 4H); ^{13}C NMR (125MHz, $CDCl_3$): δ = 17.4, 28.2, 128.3, 128.5, 131.8, 133.3, 137.1, 181.2.

This acid (1.97 g, 10 mmol) was added to a stirred suspension of $LiAlH_4$ (0.61 g, 16 mmol) in THF (48 mL) at 0 – 5 °C under an Ar atmosphere. The suspension was allowed to warm up to room temperature, followed by being stirred at the same temperature for 2 h. The mixture was quenched with water, and the resultant suspension was filtered through Celite® (No.503), using a glass filter, washing with hexane. The obtained mixture was extracted twice with AcOEt and the combined organic phase was washed with water, brine, dried (Na_2SO_4) and concentrated. The obtained crude crystal was purified by SiO_2-column chromatography (hexane/AcOEt = 3:1) to give the desired product **12** (1.75 g, 96%).

Pale yellow crystals; mp 53 – 54 °C; ^{1}H NMR (500 MHz, $CDCl_3$) δ = 0.85 (s, 4H), 1.52 (brs, 1H), 3.64 (s, 2H), 7.26-7.30 (m, 4H); ^{13}C NMR (125 MHz, $CDCl_3$) δ = 11.4, 27.4, 70.4, 128.4, 130.3, 132.2, 141.3.

1-(((1-(4-Chlorophenyl)cyclopropyl)methoxy)methyl)-3-phenoxybenzene (13) [4]

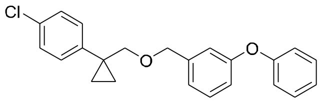

A mixture of alcohol **12** (457 mg, 2.5 mmol) and 1-benzyl-3-(bromomethyl)benzene (789 mg, 3.0 mmol) was added to a stirred suspension of NaH (60%, 120 mg, 3.0 mmol) in DMF (5 mL) at 0 – 5 °C under an Ar atmosphere, and the mixture was stirred at the same temperature for 2 h. The mixture was quenched wi th water, which was extracted twice with AcOEt. The combined organic phase was washed with water, brine, dried (Na_2SO_4) and concentrated. The obtained crude oil was purified by SiO_2-column chromatography (hexane/AcOEt = 50:1) to give the desired product **13** (836 mg, 92%).

Colorless oil; ^{1}H NMR (500 MHz, $CDCl_3$) δ = 0.85 (s, 4H), 3.51 (s, 2H), 4.44 (s, 2H), 6.90 -6.91 (m, 2H), 6.95-6.96 (m, 1H), 6.99-7.02 (m, 2H), 7.10-7.13 (m, 1H), 7.19-7.22 (m, 2H), 7.23-7.28 (m,

3H), 7.32-7.36 (m, 2H); ^{13}C NMR (125 MHz, $CDCl_3$) δ = 12.0, 24.9, 72.3, 77.4, 117.6, 117.8, 119.0, 122.0, 123.3, 128.2, 129.6, 129.7, 130.0, 131.8, 140.5, 142.0, 157.1, 157.4.

1-(((1-(4-Ethoxyphenyl)cyclopropyl)methoxy)methyl)-3-phenoxybenzene (14) [4]

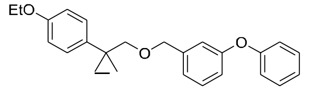

A mixture of ether **13** (364 mg, 1.0 mmol), $Pd_2(dba)_3$ (18.3 mg, 0.02 mmol), *t*Bu-XPhos (34.1 mg, 0.08 mmol), and KOH (280 mg, 5.0 mmol) in 1,4-dioxane/water (1:1, 2 mL) was stirred at 100 – 105 °C for 16 h under an Ar atmosphere. The mixture was cool down to room temperature, and quenched with 6 M-HCl aq., which was extracted twice with AcOEt. The combined organic phase was washed with water, brine, dried (Na_2SO_4) and concentrated. The obtained crude oil was purified by SiO_2-column chromatography (hexane/AcOEt = 5:1) to give the desired phenol intermediate (325 mg, 94%).

Pale yellow oil; ^{1}H NMR (500 MH, $CDCl_3$): δ = 0.76-0.83 (m, 4H), 3.50 (s, 2H), 4.45 (s, 2H), 6.65-6.66 (m, 2H), 6.89-6.91 (m, 2H), 6.96-7.00 (m, 3H), 7.09-7.12 (m, 1H), 7.17-7.19 (m, 2H), 7.23-7.27 (m, 1H), 7.32-7.35 (m, 2H); ^{13}C NMR (125 MHz, $CDCl_3$): δ = 11.5, 24.8, 72.2, 77.9, 114.9, 117.7, 117.8. 118.9, 122.1, 123.2, 129.6, 129.7, 130.0, 135.4, 140.6, 154.1, 157.2, 157.3; IR (neat): ν_{max} = 3377, 3076, 2860, 1584, 1518, 1487, 1254, 1213, 1072, 910, 835, 771, 738, 692 cm^{-1}; HRMS (ESI): *m/z* calcd for $C_{10}H_{16}O_3$ $[M + Na]^+$ 369.1467; found: 369.1471.

K_2CO_3 (100 mg, 0.72 mmol) was added to a stirred solution of this phenol intermediate (208 mg, 0.6 mmol) in DMF (1.2 mL) at 0 – 5 °C under an Ar atmosphere. Then, EtI (0.14 mL, 3.0 eq.) was added to the mixture, which was stirred at the room temperature for 14 h. The mixture was quenched with water, which was extracted twice with AcOEt. The combined organic phase was washed with water, brine, dried (Na_2SO_4) and concentrated. The obtained crude oil was purified by SiO_2-column chromatography (hexane/AcOEt = 50:1) to give the desired product **14** (190 mg, 85%).

Pale yellow oil; ^{1}H NMR (500 MH, $CDCl_3$): δ = 0.78-0.83 (m, 4H), 1.39 (t, *J* = 6.9 Hz, 3H), 3.50 (s, 2H), 3.99 (q, *J* = 6.9 Hz, 2H), 4.44 (s, 2H), 6.77-6.80 (m, 2H), 6.88-6.91 (m, 2H), 6.96-7.01 (m, 3H), 7.09-7.12 (m, 1H), 7.23-7.27 (m, 3H), 7.32-7.36 (m, 2H); ^{13}C NMR (125 MHz, $CDCl_3$) δ = 1.0, 11.5, 14.9, 24.8, 63.3, 72.2, 77.8, 114.0, 117.7, 117.7, 118.9, 122.1, 123.2, 129.5, 129.7, 129.8, 135.4, 140.7, 157.1, 157.3, 157.3.

Methyl 2-(4-chlorophenyl)-2-methylpropanoate (16) [5]

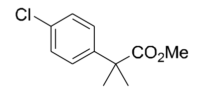

Methyl 2-(4-chlorophenyl)acetate (**15**; 1.85 g, 10 mmol) was added to a stirred suspension of NaH (60%, 0.88 g, 22 mmol) in DMF (20 mL) at 0 – 5 °C under an Ar atmosphere, followed by being stirred at the same temperature for 0.5 h. MeI (2.5 mL, 40 mmol) was added to the mixture, followed by being stirred at the same temperature for 0.5 h. The mixture stirred at the room temperature for 15 h. The mixture was quenched with water and extracted twice with AcOEt. The combined organic phase was washed with water, brine, dried (Na_2SO_4) and concentrated. The obtained crude oil was purified by SiO_2-column chromatography (hexane/AcOEt = 50:1) to give the desired product **16** (1.89 g, 89%).

Pale yellow oil; ^{1}H NMR (500 MHz, $CDCl_3$): δ = 1.56 (s, 6H), 3.65 (s, 3H), 7.26-7.30 (m, 4H); ^{13}C NMR (125 MHz, $CDCl_3$) δ = 26.4, 46.1, 52.3, 127.1, 128.5, 132.6, 143.1, 176.8.

2-(4-Chlorophenyl)-2-methylpropan-1-ol (17) [6]

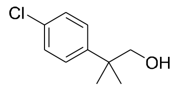

Following the similar procedure for the preparation of alcohol **12**, the reaction of ester **16** (1.32g, 6.2 mmol) with $LiAlH_4$ (374 mg, 9.9 mmol) in THF (30 mL) and the successive purification by SiO_2-column chromatography (hexane/AcOEt = 2:1) gave the desired product **8** (1.09 g, 95%) .

Colorless crystals: mp 48 – 49 °C [lit.[6] 46 – 48 °C]; ^{1}H NMR (500 MHz, $CDCl_3$): δ = 1.19-1.27 (brs, 1H), 1.32 (s, 6H), 3.60 (s, 2H), 7.30-7.34 (m, 4H); ^{13}C NMR (125 MHz, $CDCl_3$) δ = 25.2, 39.8, 72.8, 127.7, 128.4, 131.9, 145.0.

1-((2-(4-Chlorophenyl)-2-methylpropoxy)methyl)-3-phenoxybenzene (18) [6]

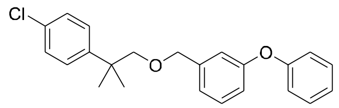

Following the similar procedure for the preparation of ether **13** [4], the reaction of alcohol **17** (462 mg, 2.5 mmol), with 1-benzyl-3-(bromomethyl)benzene (789 mg, 3.0 mmol) , and NaH (60%, 120 mg, 3.0 mmol) and the successive purification by SiO_2-column chromatography (hexane/AcOEt = 50:1) gave the desired product **18** (927 mg, 99%).

Colorless oil; ^{1}H NMR (500 MHz, $CDCl_3$) δ = 1.30 (s, 6H), 3.41 (s, 2H), 4.43 (s, 2H), 6.90 -6.92

(m, 2H), 6.95-6.96 (m, 1H), 7.00-7.02 (m, 2H), 7.10-7.13 (m, 1H), 7.21-7.29 (m, 5H), 7.32-7.36 (m, 2H); ^{13}C NMR (125 MHz, $CDCl_3$) δ = 26.0, 38.9, 72.7, 79.8, 117.4, 117.7, 119.0, 121.9, 123.3, 127.6, 128.0, 129.5, 129.7, 131.6, 140.7, 146.0, 157.1, 157.4.

Etofenprox [6]

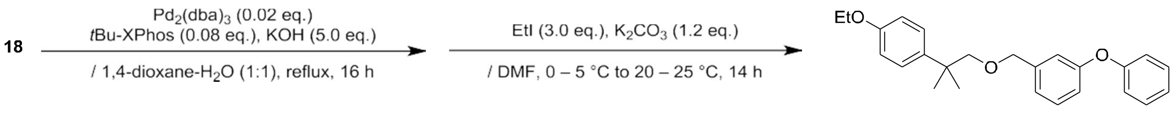

Etofenprox

Following the similar procedure for the preparation of ether **14** the reaction of ether **18** (367 mg, 1.0 mmol) with $Pd_2(dba)_3$ (18.3 mg, 0.02 mmol), *t*Bu-XPhos (34.1 mg, 0.08 mmol), and KOH (280 mg, 5.0 mmol), and the successive purification by SiO_2-column chromatography (hexane/AcOEt = 5:1) gave the desired phenol intermediate (291 mg, 84%).

Colorless oil; ^{1}H NMR (500 MHz, $CDCl_3$): δ = 1.30 (s, 6H), 3.40 (s, 2H), 4.40 (s, 2H), 6.72 -6.75 (m, 2H), 6.90-6.92 (m, 2H), 6.97-7.02 (m, 3H), 7.09-7.13 (m, 1H), 7.21-7.28 (m, 3H), 7.32-7.36 (m, 2H); ^{13}C NMR (125 MHz, $CDCl_3$): δ = 26.3, 38.6, 72.9, 80.5, 115.0, 117.7, 117.9, 119.1, 122.2, 123.4, 127.2, 129.7, 129.9, 139.2, 141.0, 154.1, 157.3, 157.4

Following the similar procedure for the preparation of ether **13**, the reaction of phenol **14** (209 mg, 0.6 mmol) with K_2CO_3 (100 mg, 0.72 mmol) and EtI (0.14 mL, 3.0 eq.), and the successive purification by SiO_2-column chromatography (hexane/AcOEt = 50:1) gave the desired Etofenprox (195 mg, 86%).

Colorless oil; ^{1}H NMR (500 MH, $CDCl_3$): δ = 1.30 (s, 6H), 1.38 (t, *J* = 6.9 Hz, 3H), 3.40 (s, 2H), 3.98 (q, *J* = 6.9 Hz, 2H), 4.43 (s, 2H), 6.79-6.83 (m, 2H), 6.88-6.93 (m, 2H), 6.97-7.01 (m, 3H), 7.08-7.12 (m, 1H), 7.24-7.27 (m, 3H), 7.30-7.34 (m, 2H); ^{13}C NMR (125 MH, $CDCl_3$): δ = 14.9, 26.1, 38.4, 63.2, 72.7, 80.2, 113.9, 117.5, 117.6, 118.9, 121.9, 123.2, 127.0, 129.5, 129.7, 139.4, 141.0, 156.9.

[1] Harnisch, J.; Szeimies, G. *Chem. Ber.* **1979**, *112*, 3914–3933.

[2] Reaction Conditions: Barbasiewicz, M.; Ma rcinial, K.; Fedoryński, M. *Tetrahedron Lett.* **2006**, *47*, 3870–3874.

[3] Egger, H. *Belg.* **1985**, BE 900594 A1 19850318.

[4] Nishida, S.; Matsuo, N.; Tsushima, K.; Hatakoshi, M.; Hirano, M. *Eur. Pat. Appl.* **1983** EP 94085 A1 19831116.

[5] Kolbe, A.; Liebisch, H.W.; Miersch, O.; Schreiber, K.; Schuette, H. R.; Schulze, C.; Sembdner, G.; Unverricht, A.; Voigtlaender, D. *Ger. Offen.* **1975**, DE 2458142 A1 19750717.

[6] Nakatani, K.; Numata, S.; Inoue, T.; Kodaka, K.; Ishii, T.; Toyama, T.; Ta chibana, H.; Udagawa, T.; Gohbara, M. *Fr. Demande* **1981**, FR 2481695 A1 19811106.
