# Supplementary figures and images for: Synthesis and Stereostructure-Activity Relationship of Novel Pyrethroids Possessing Two Asymmetric Centers on a Cyclopropane Ring
Source: Molecules. 2019 Mar 14;24(6):1023. doi: 10.3390/molecules24061023 (PMC6471473; doi:10.3390/molecules24061023)

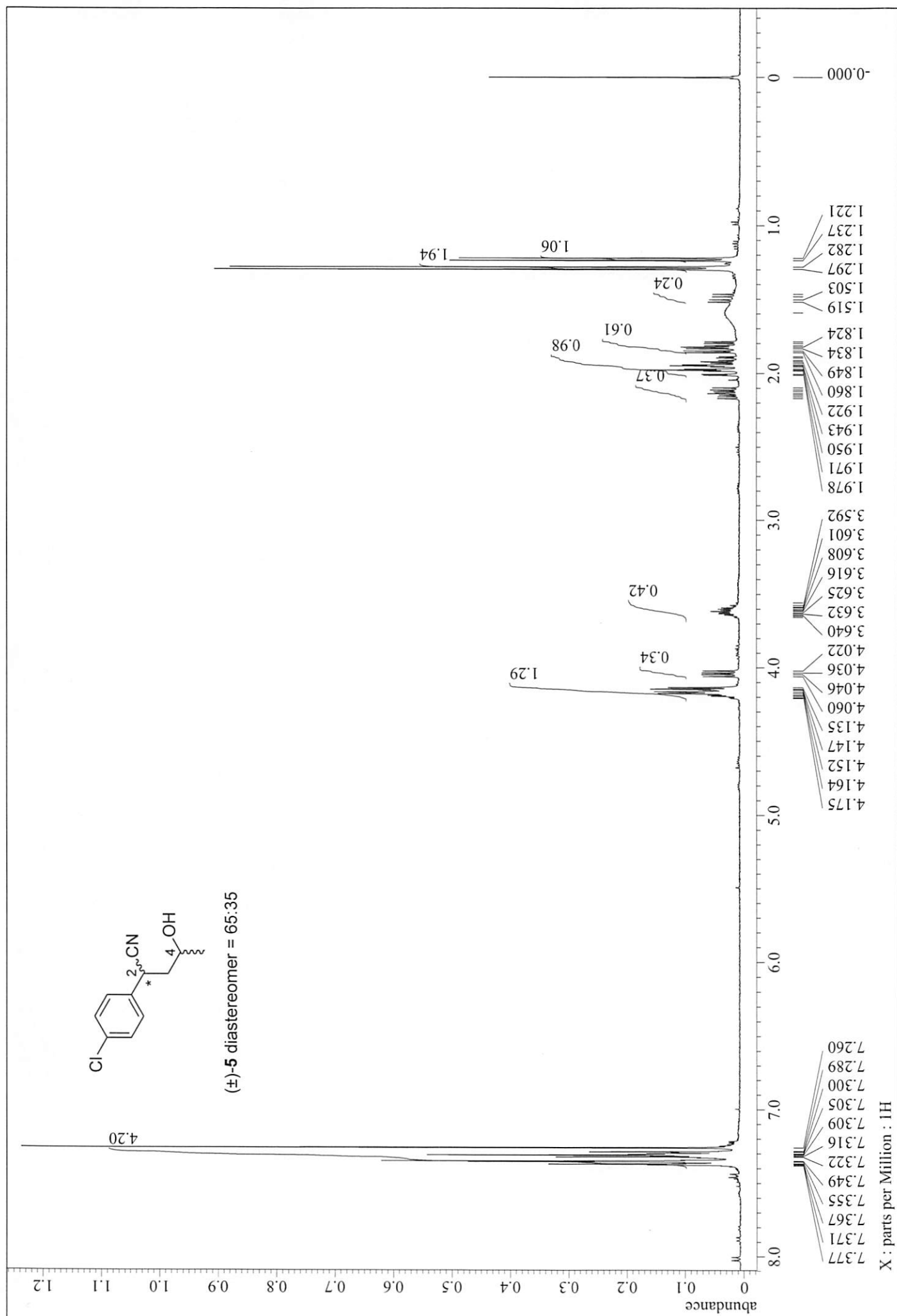

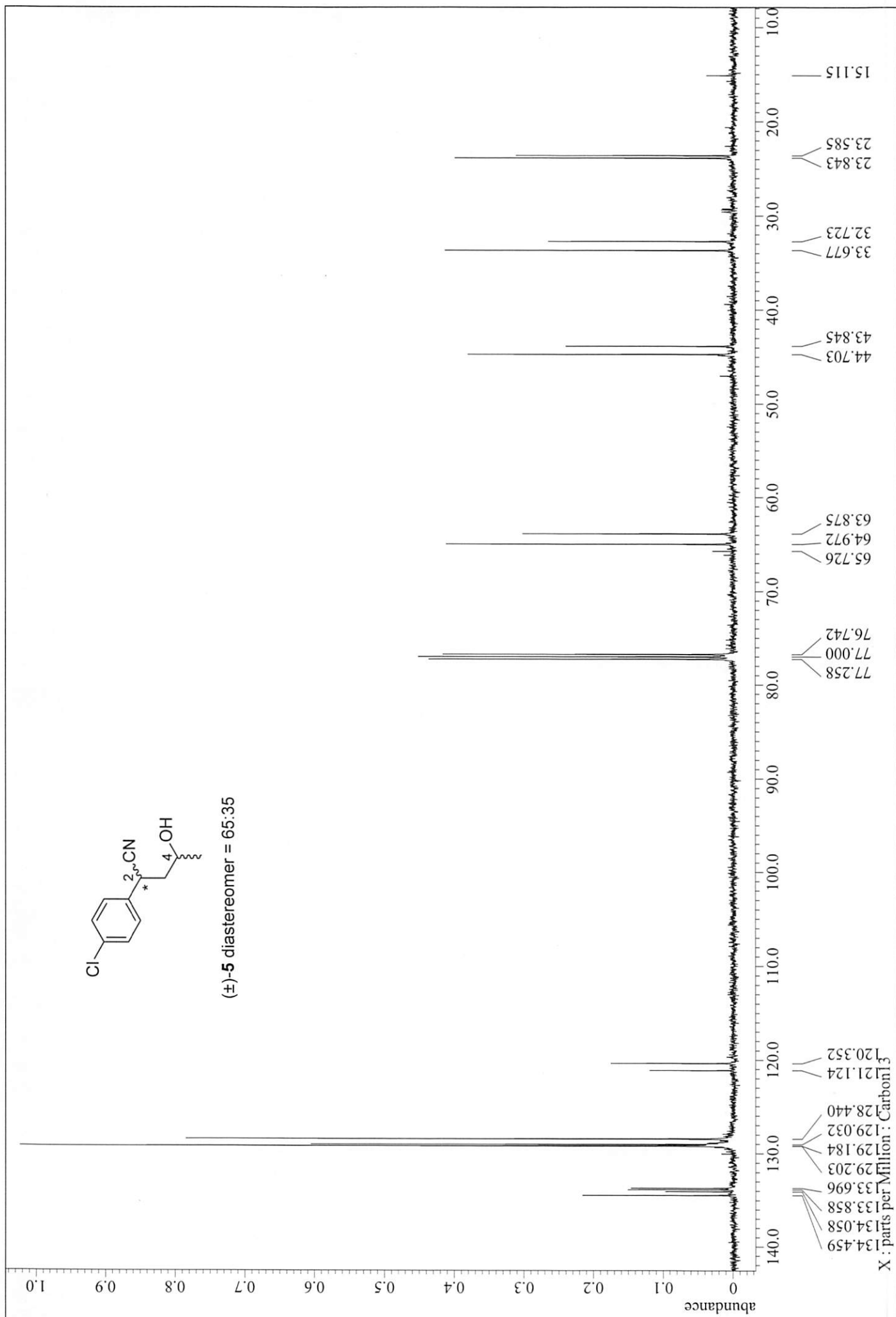

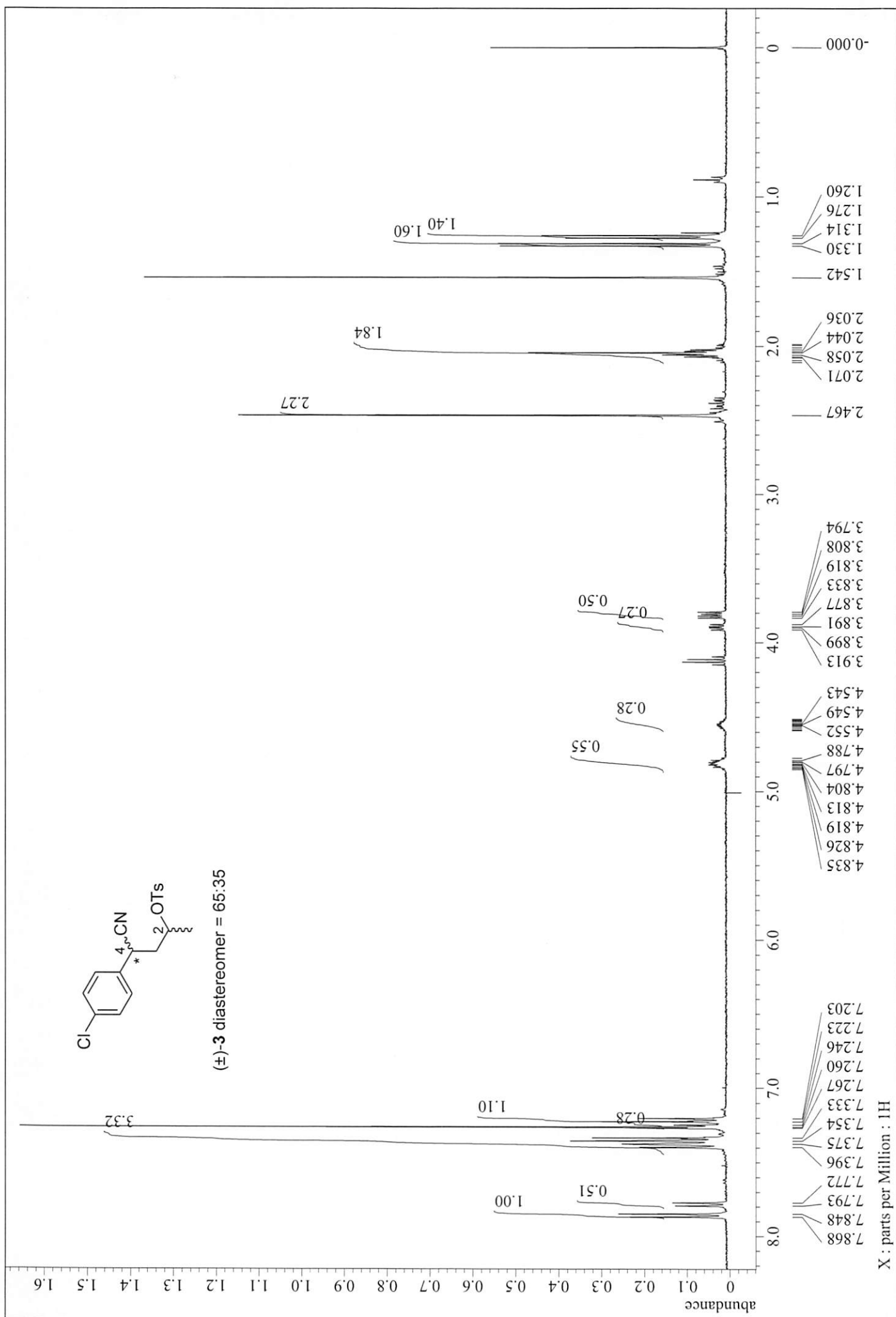

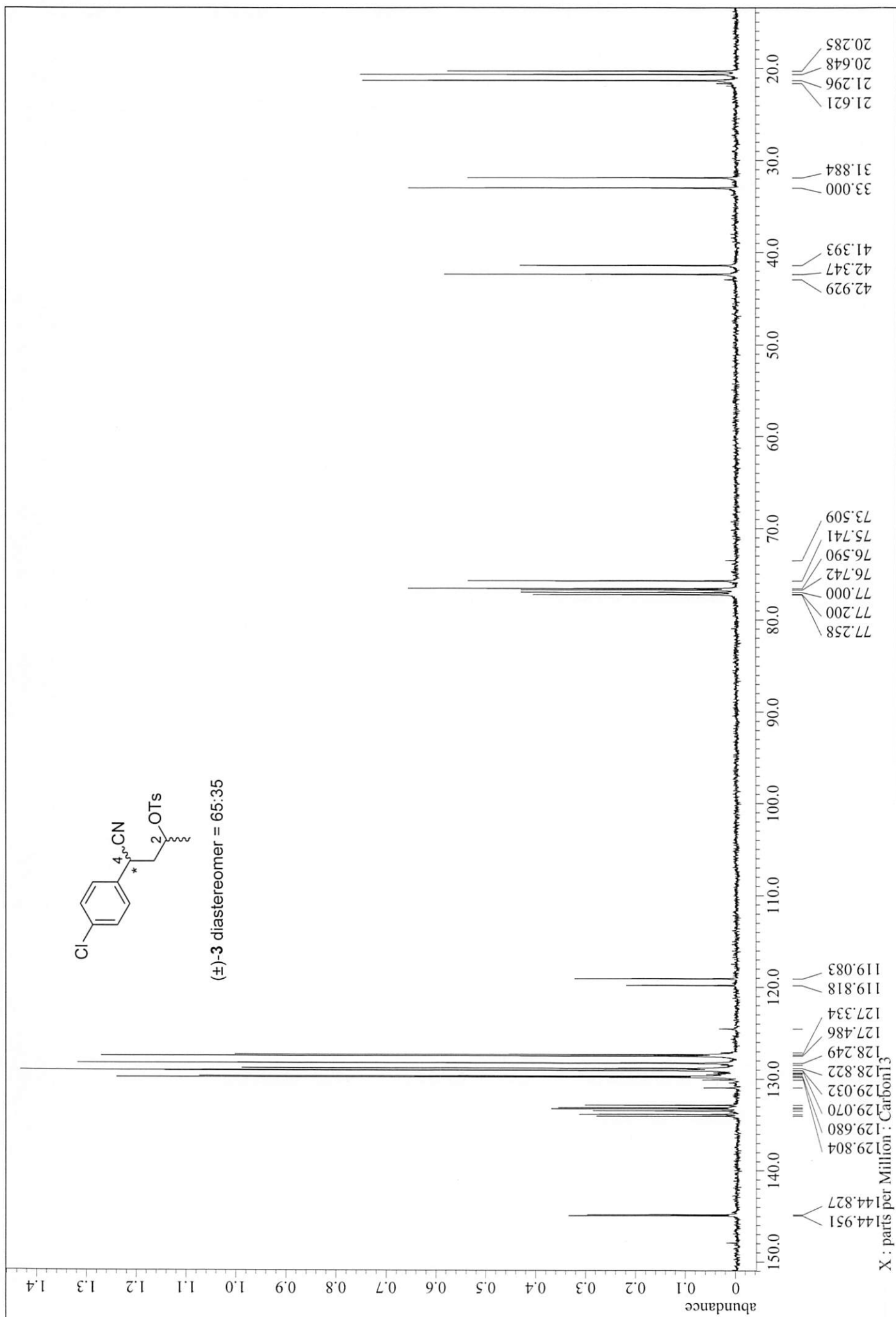

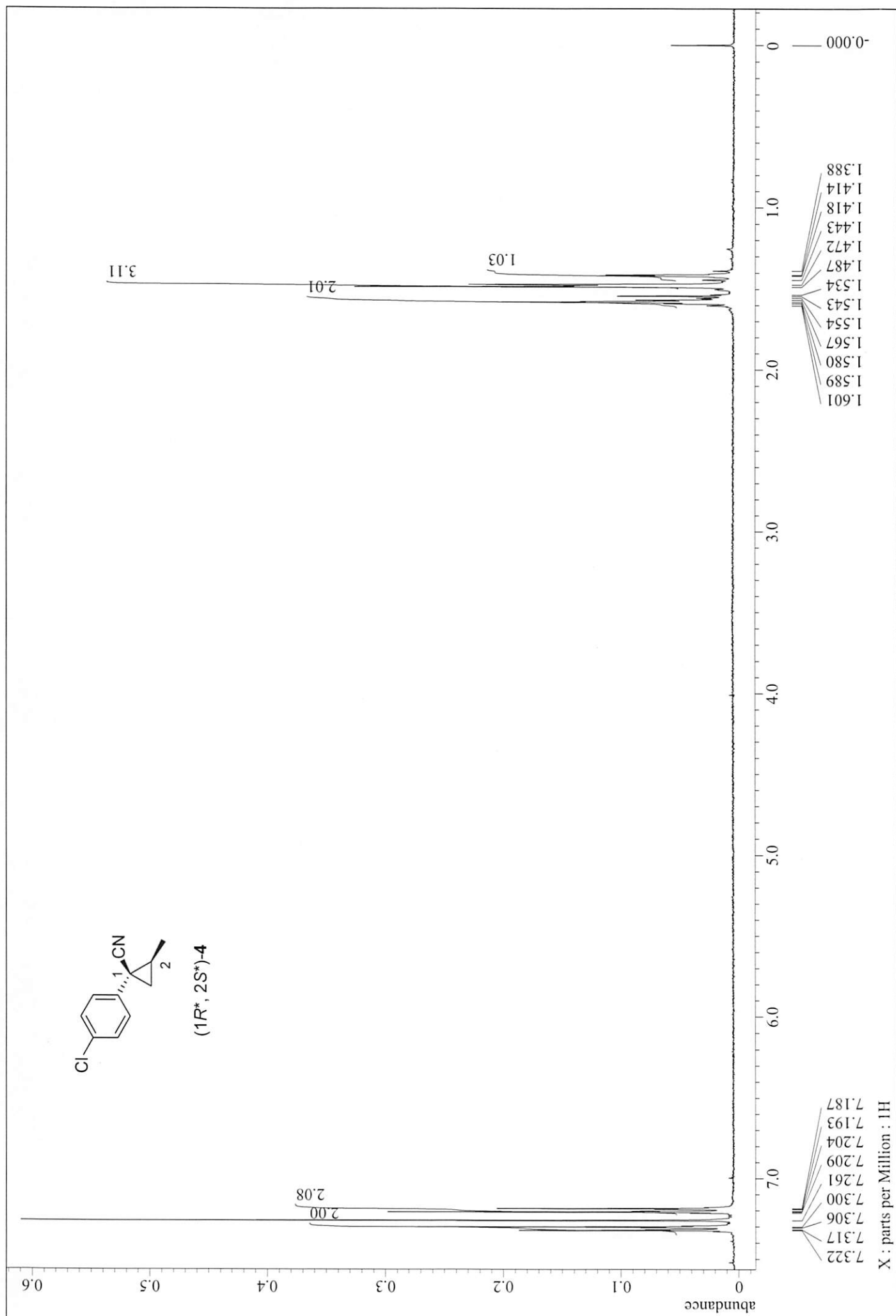

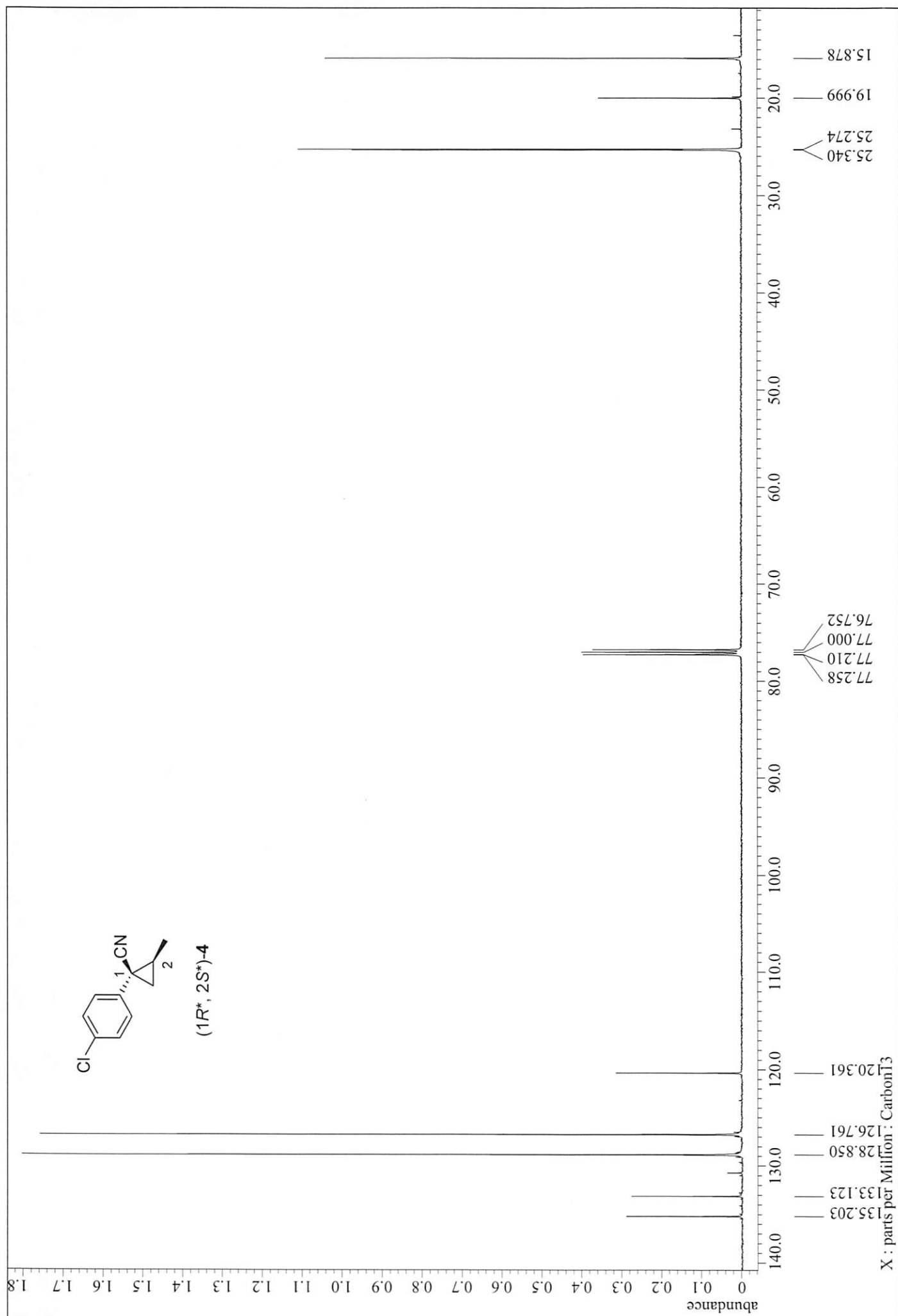

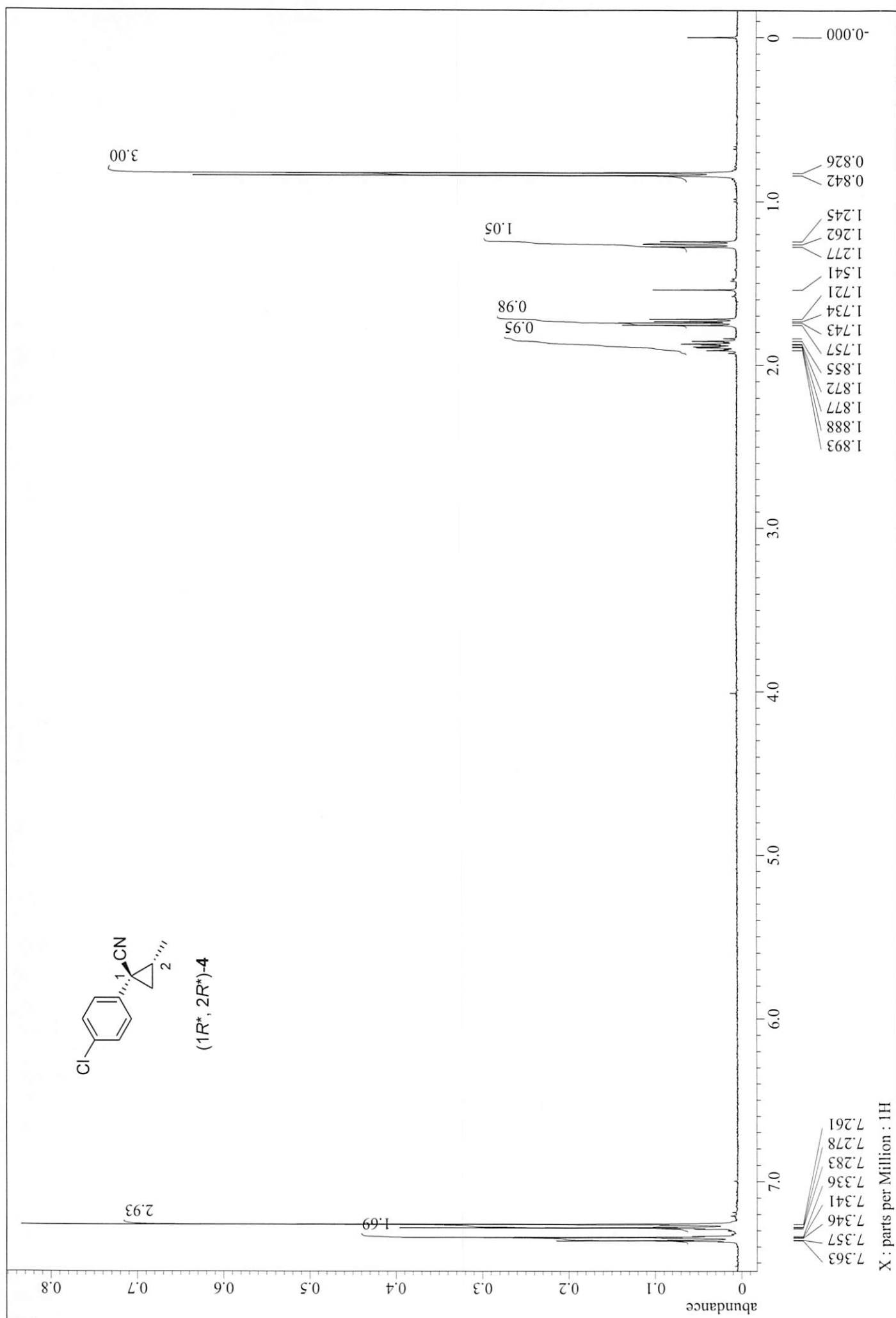

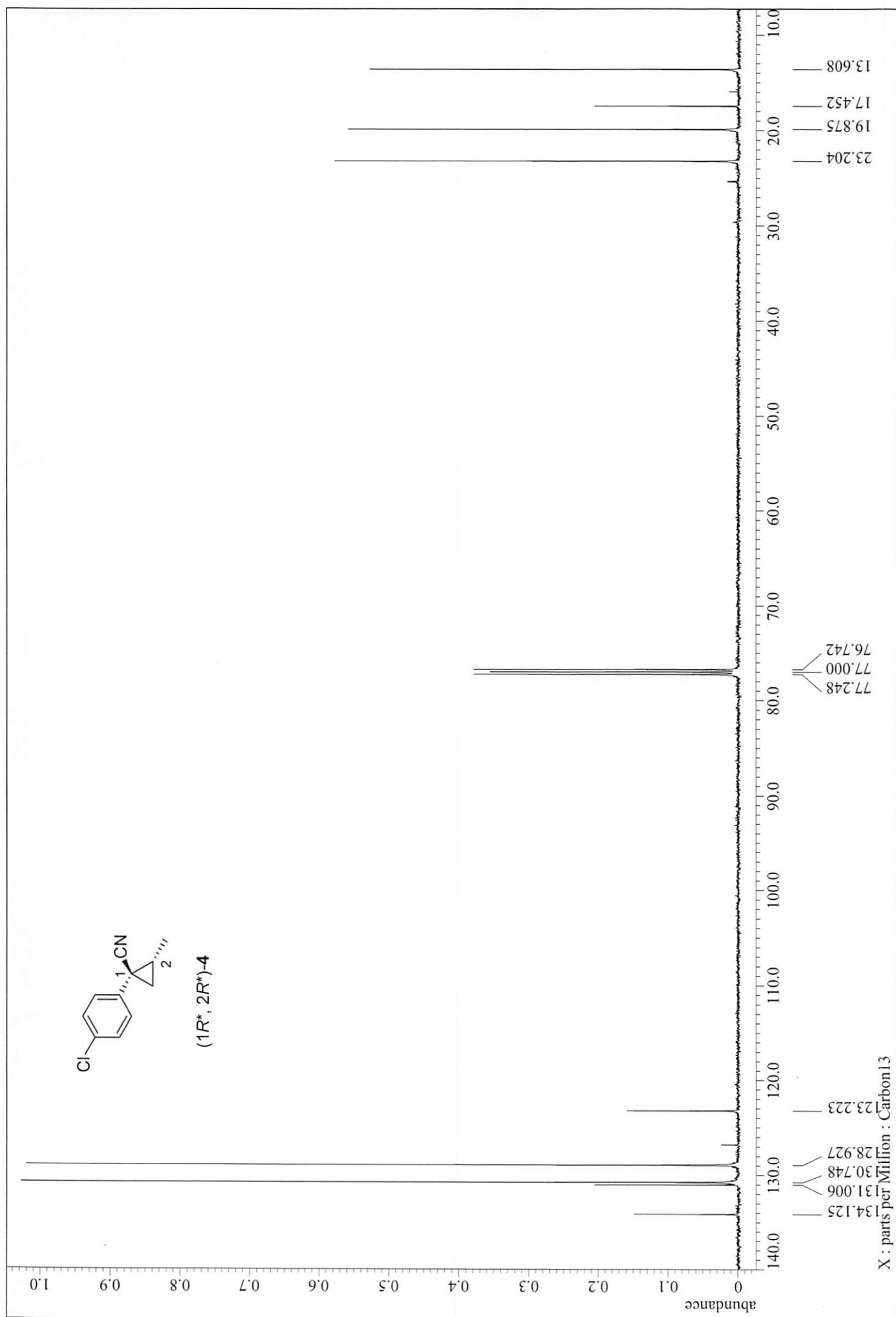

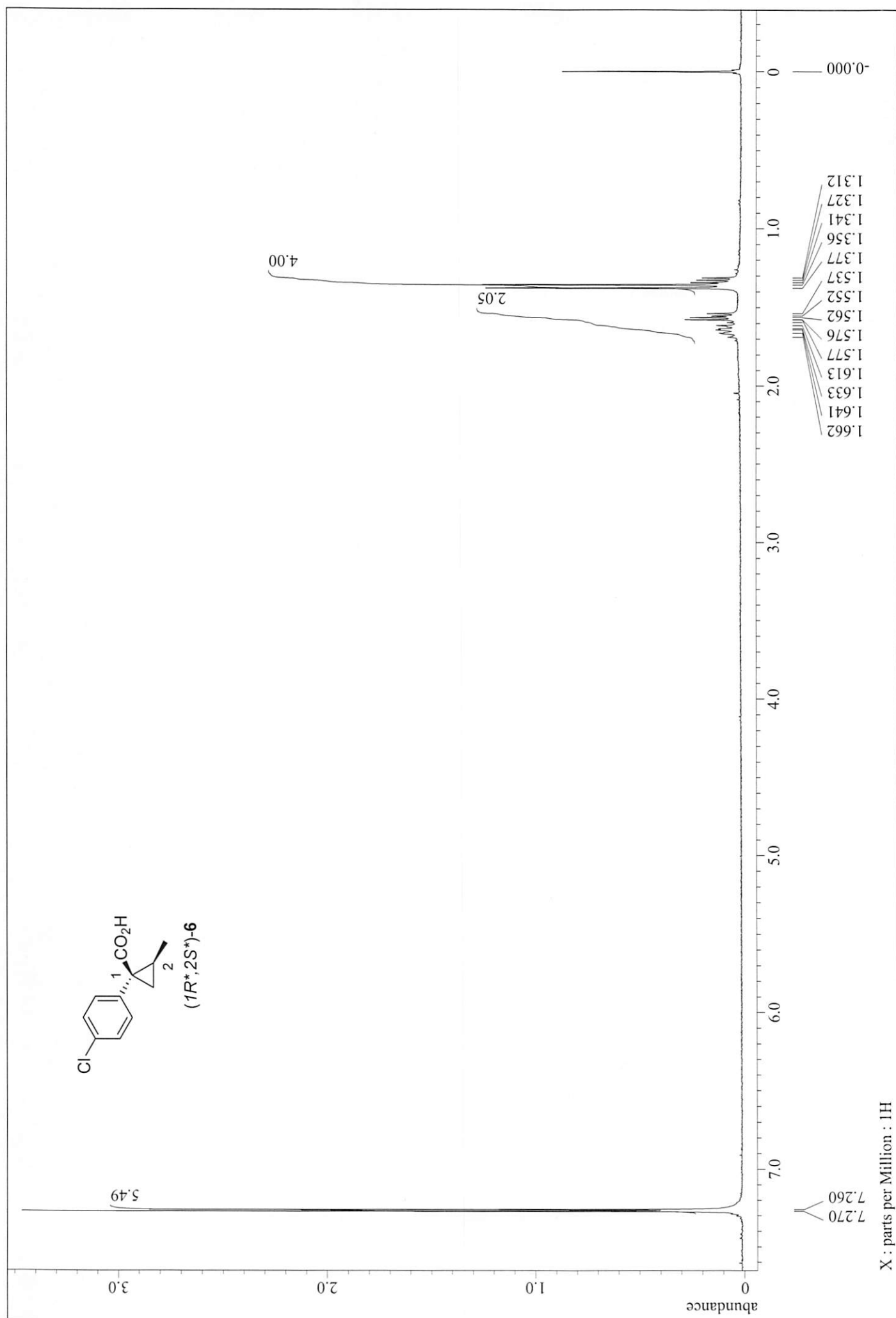

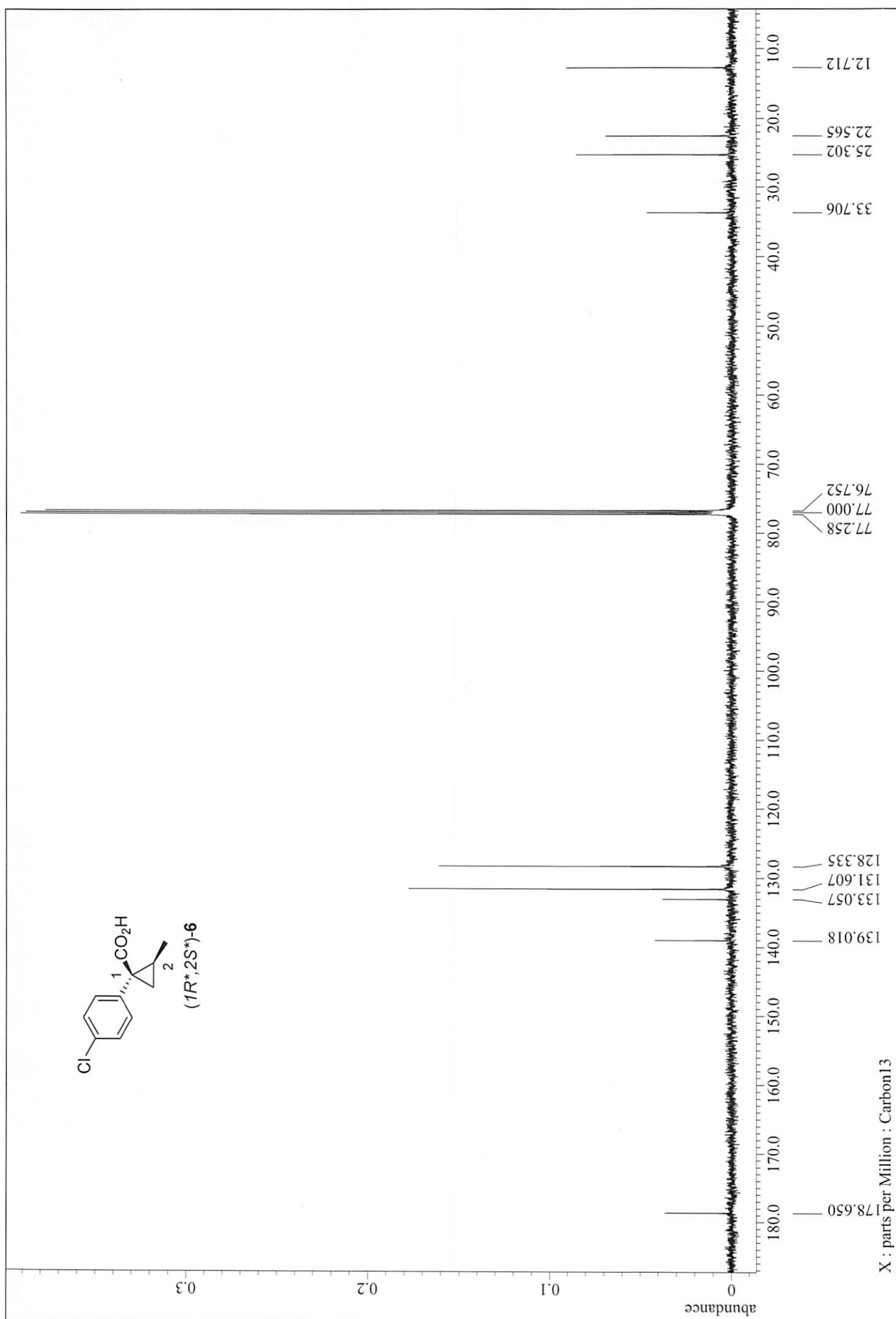

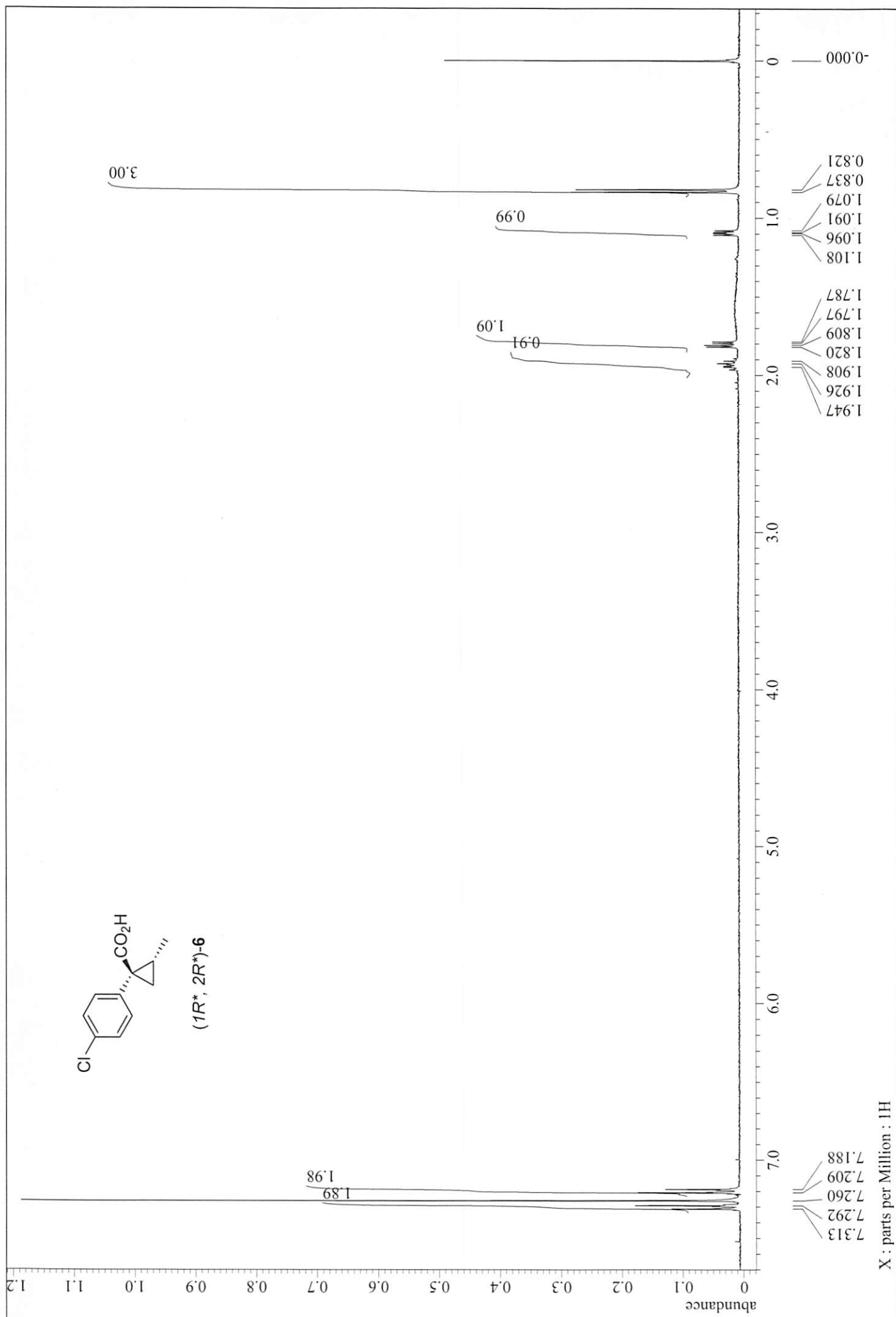

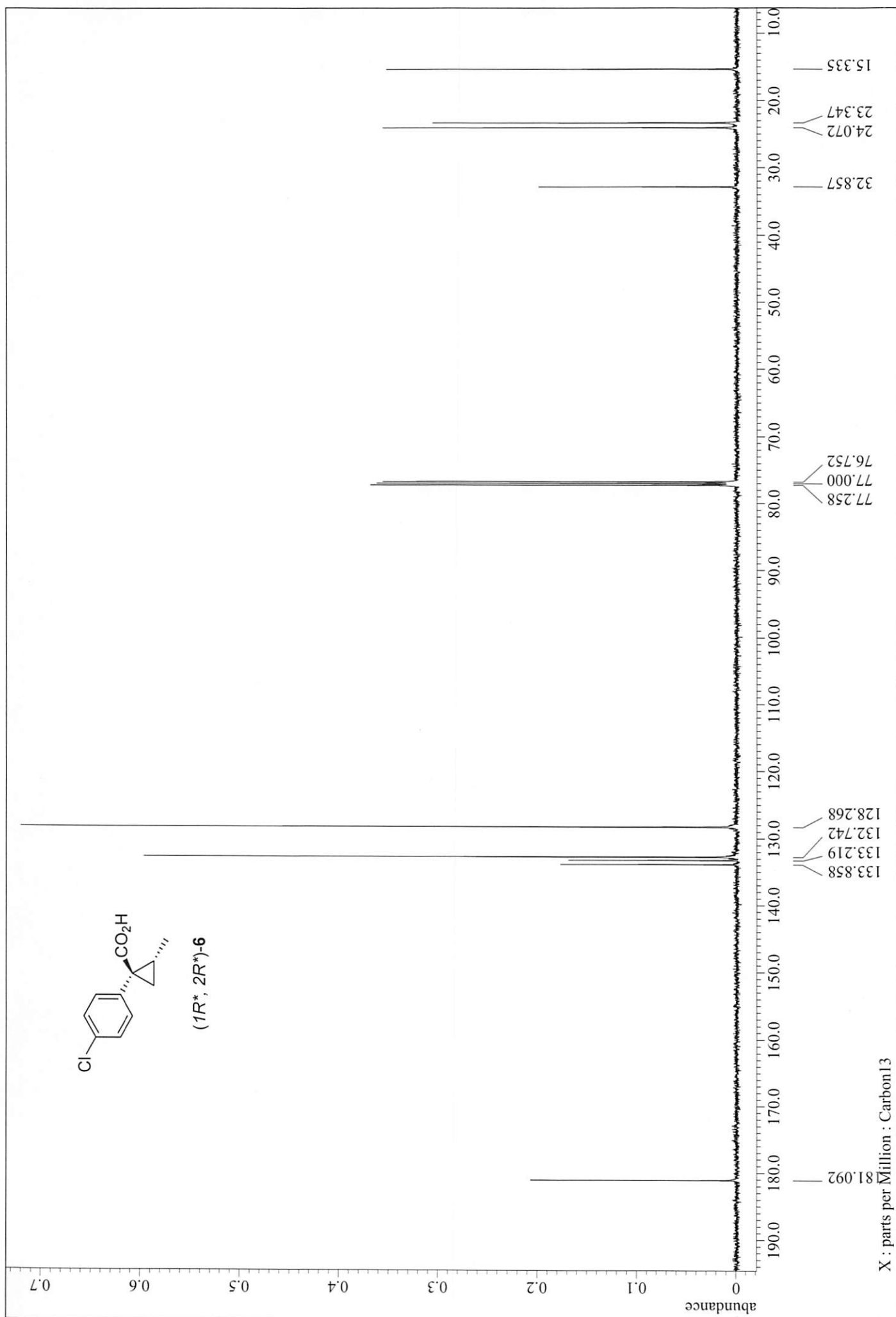

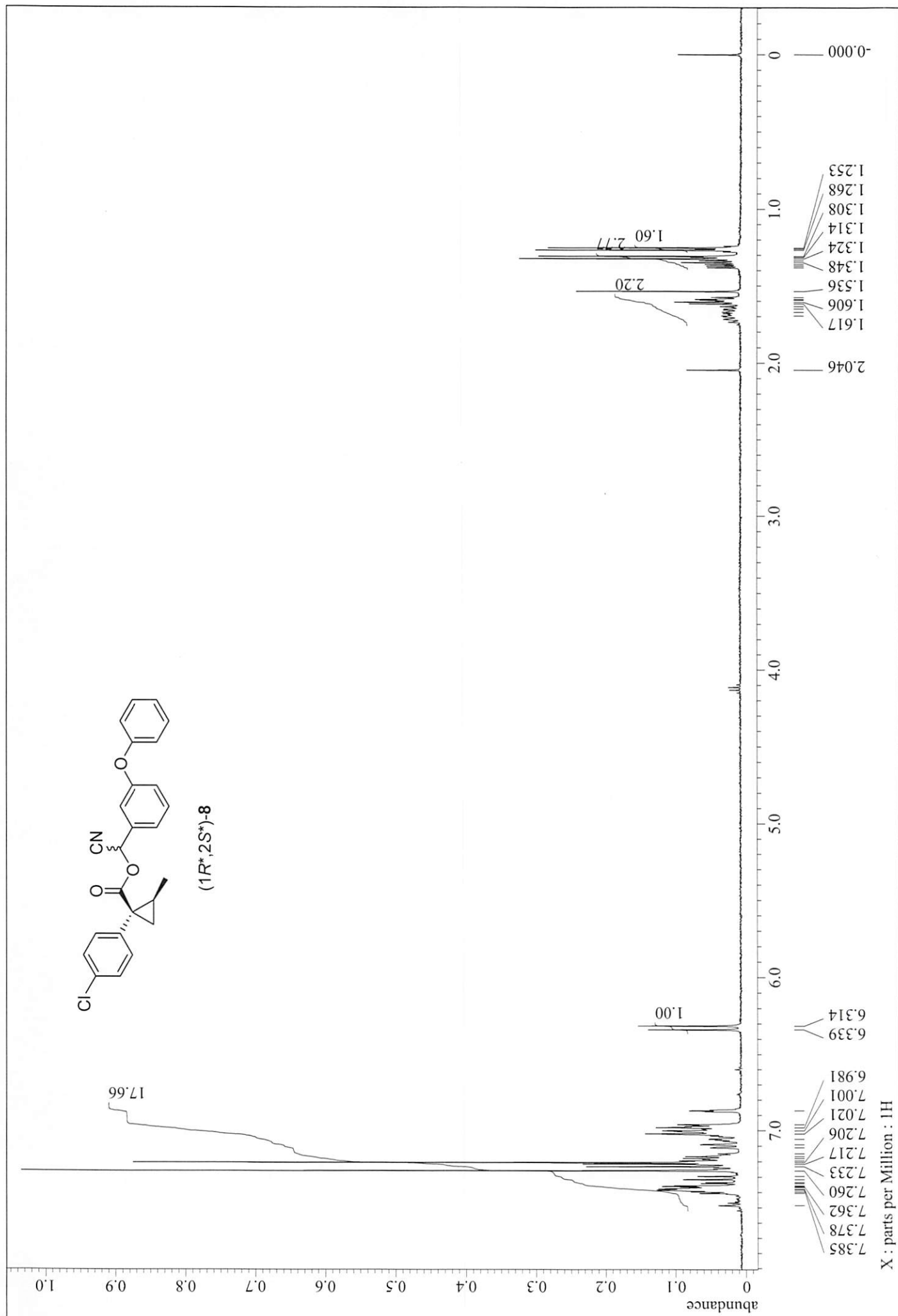

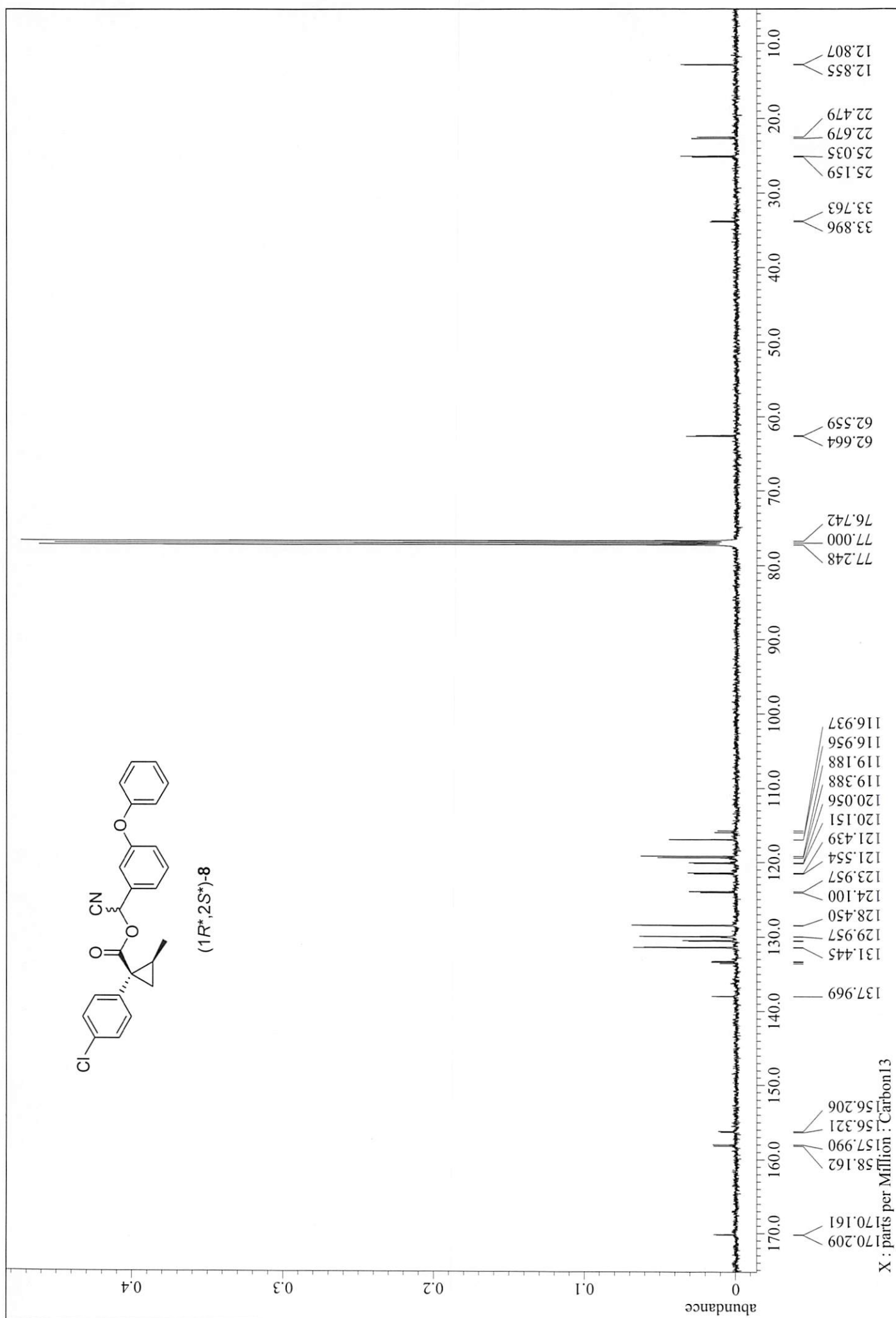

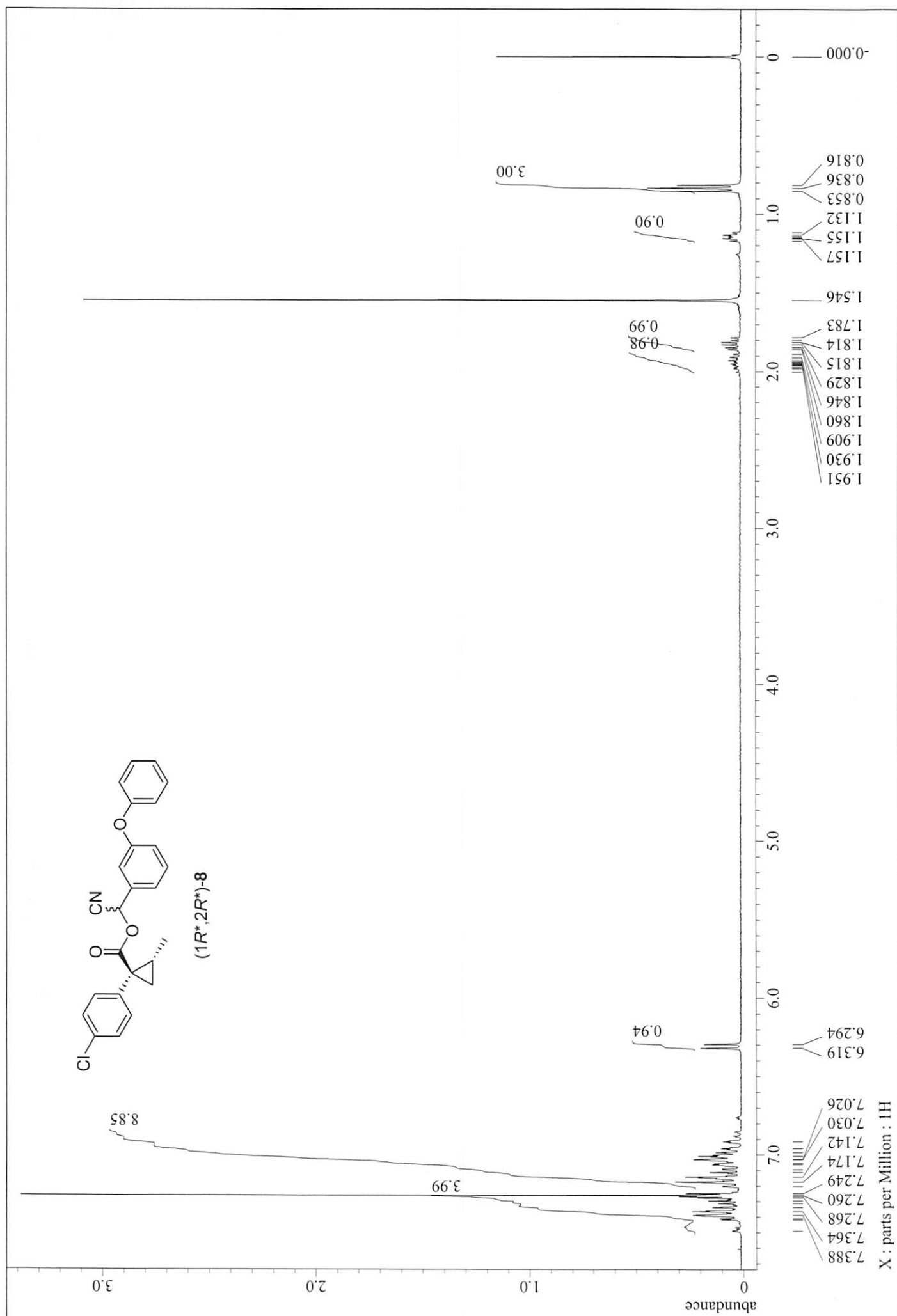

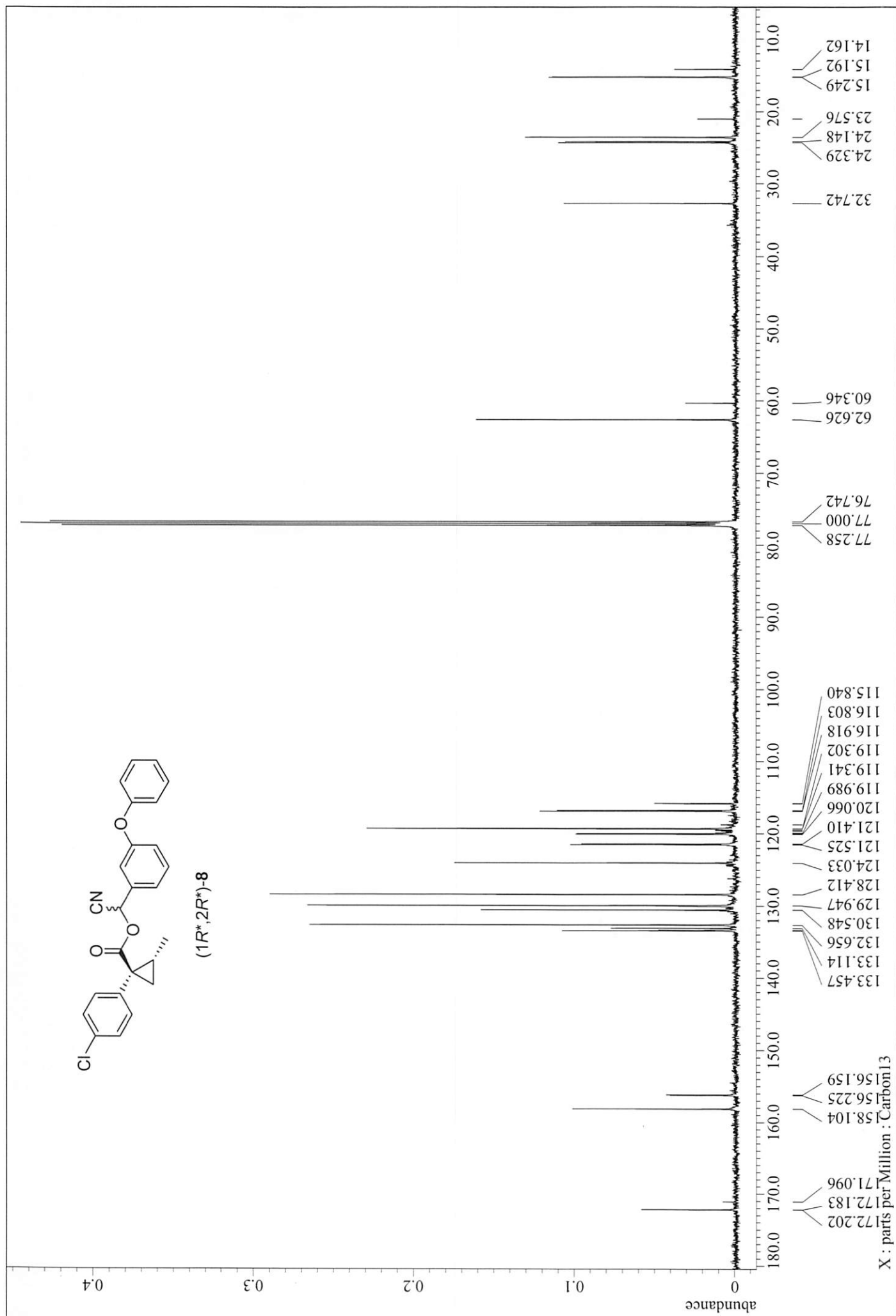

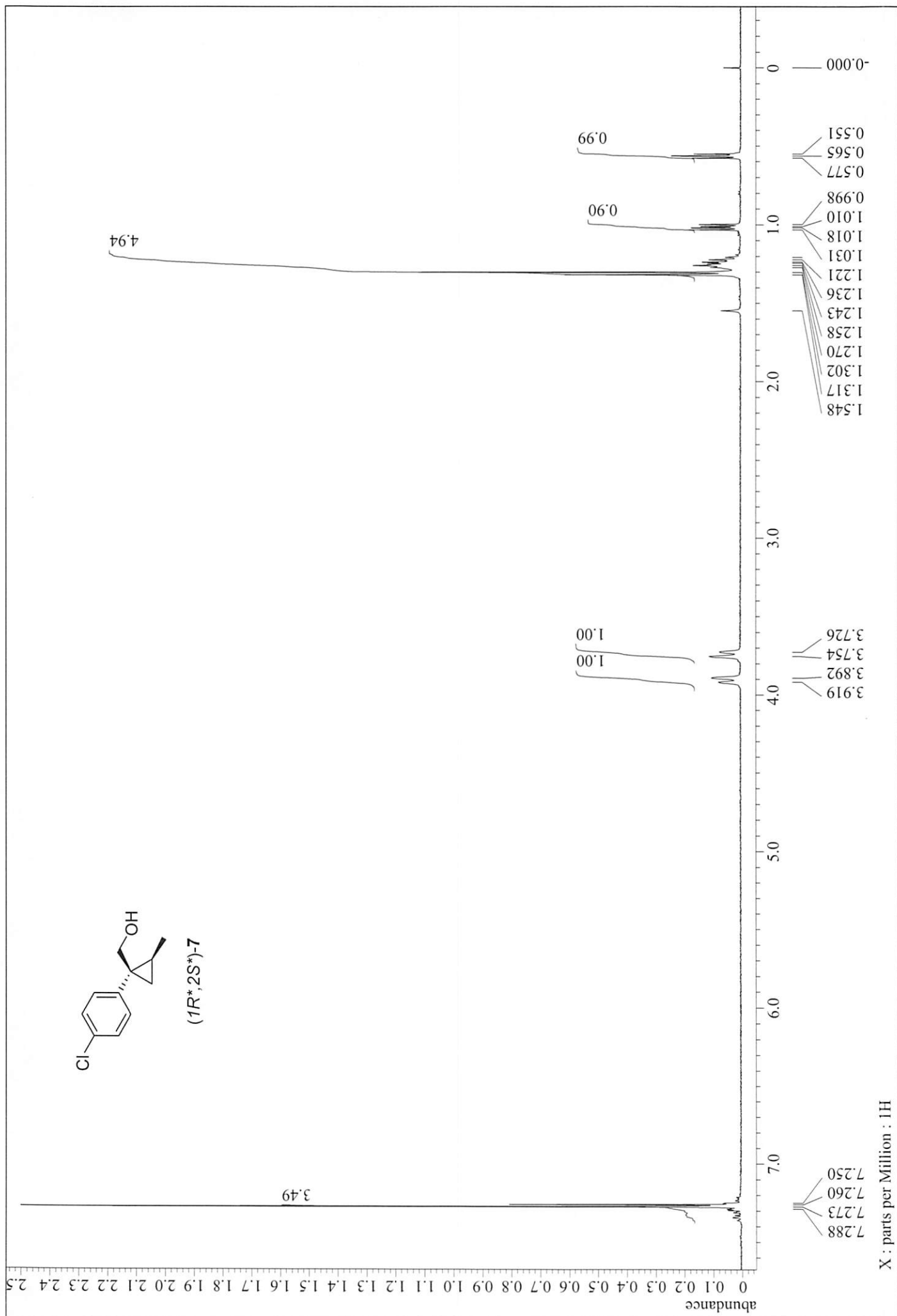

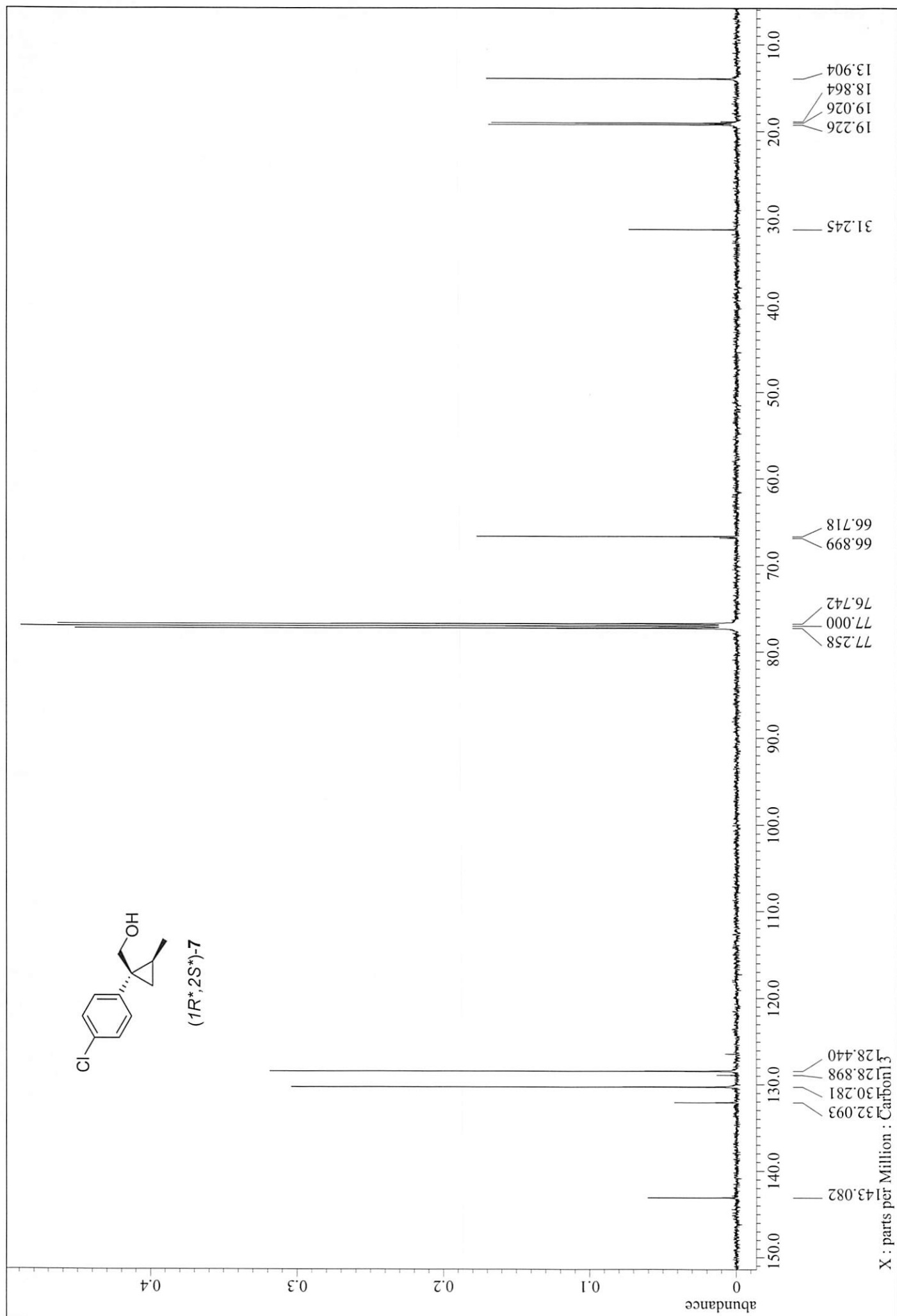

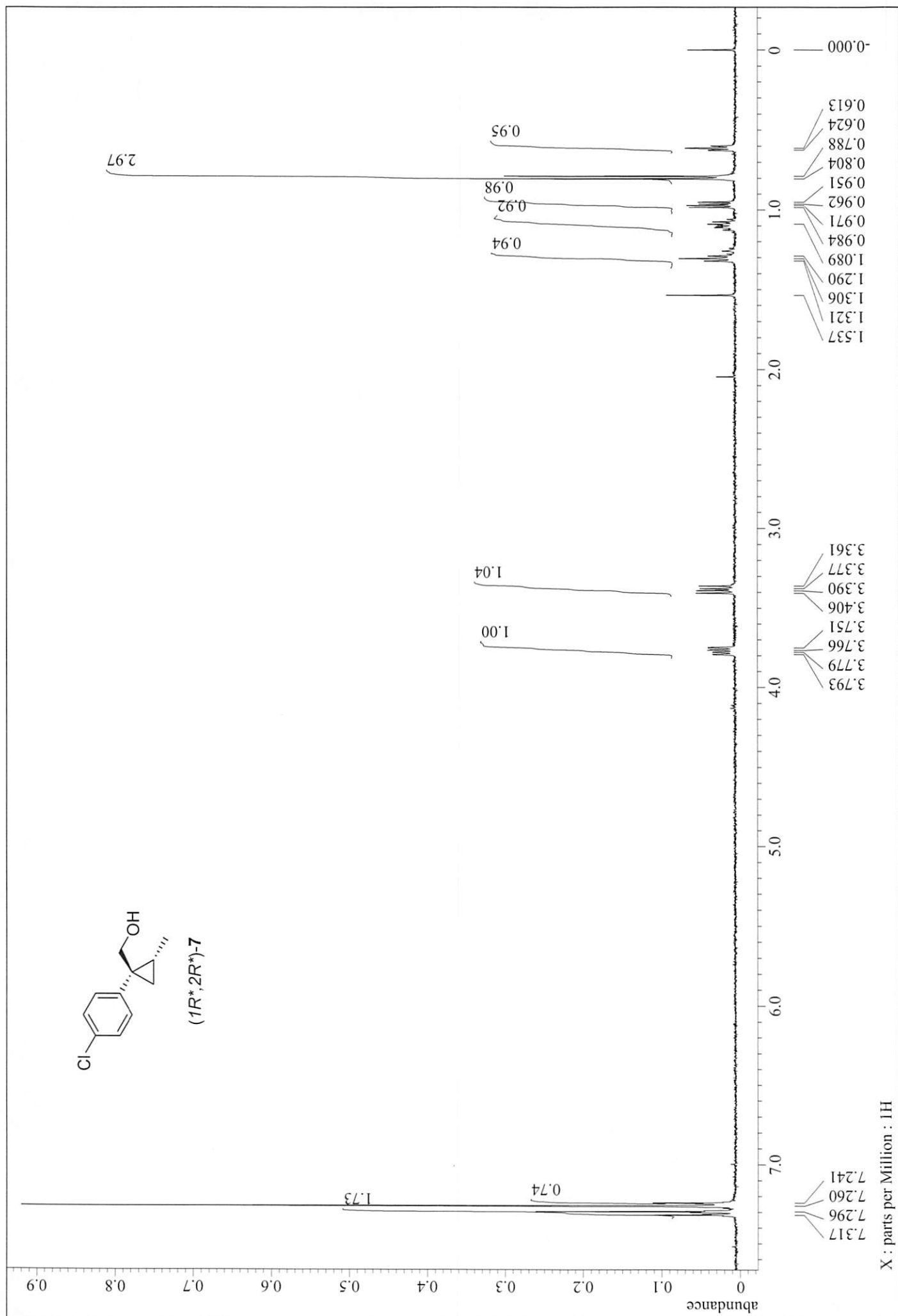

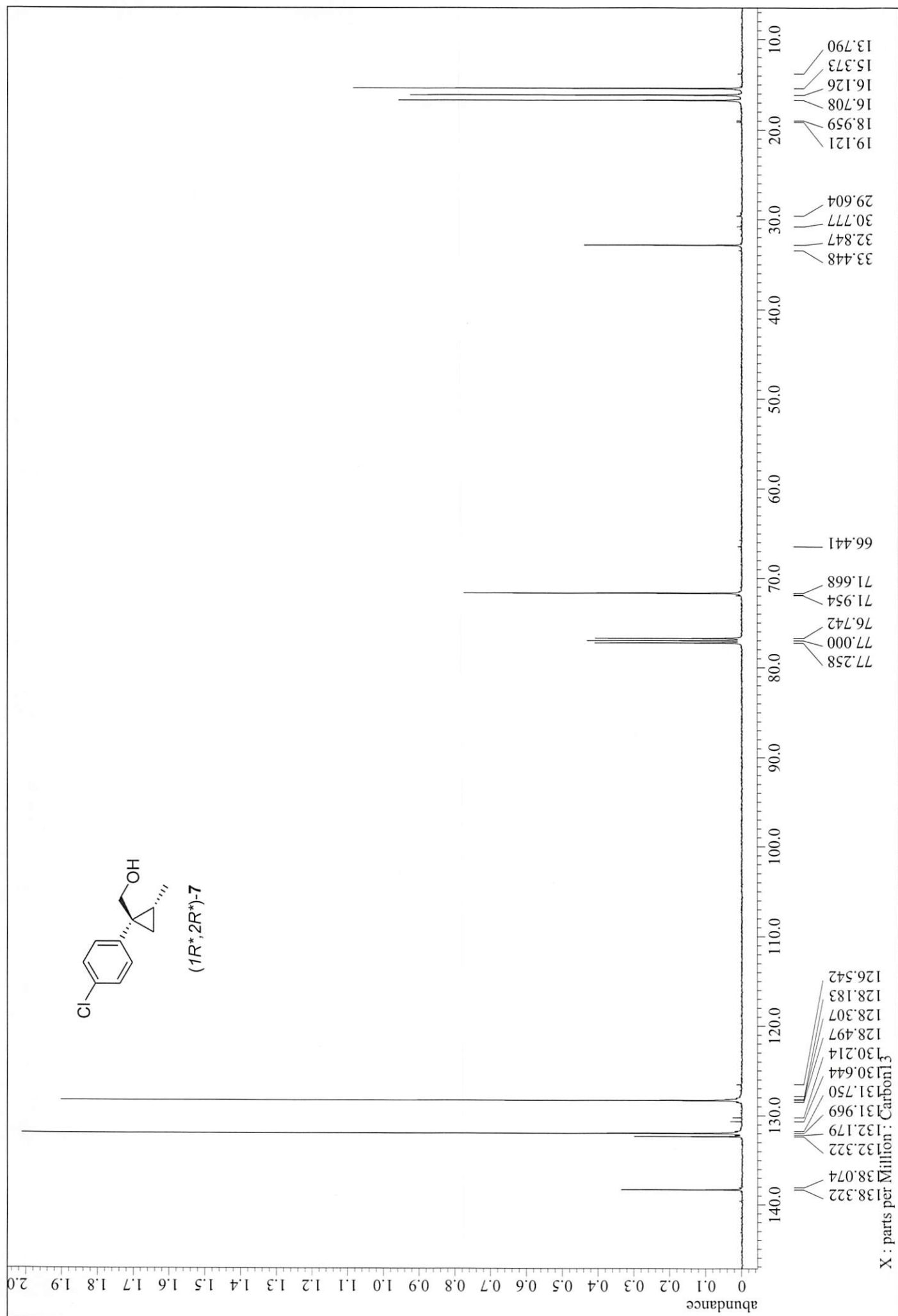

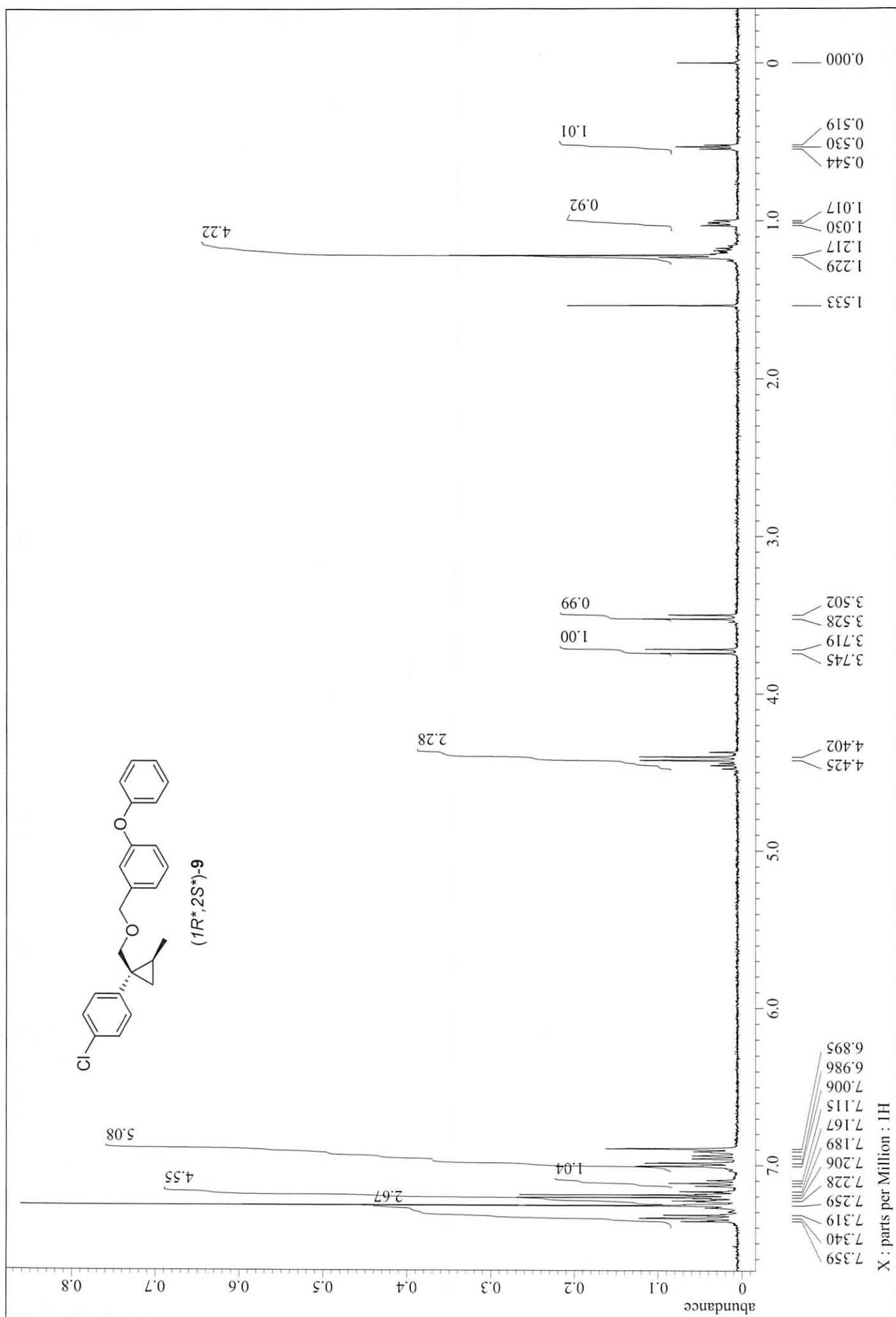

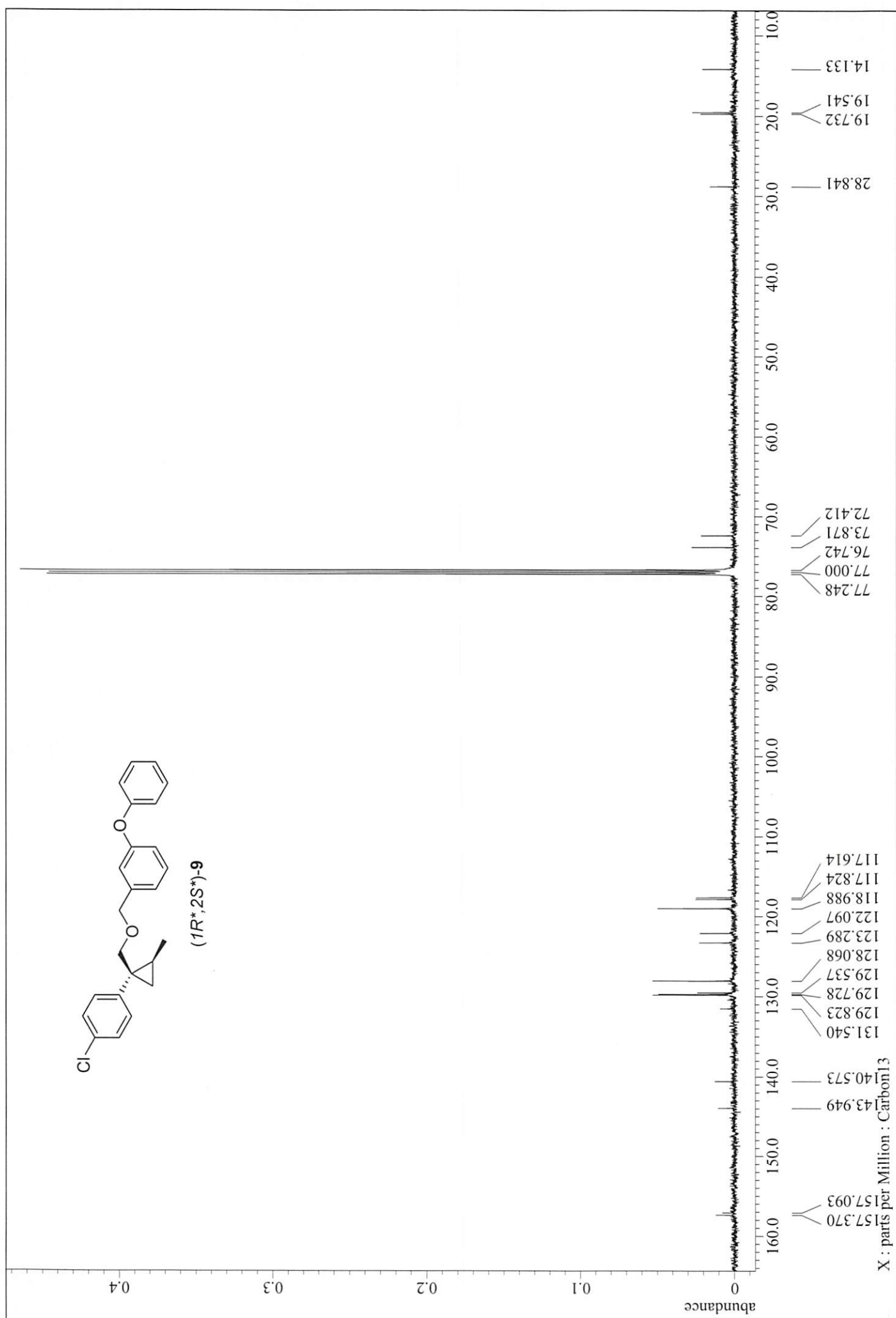

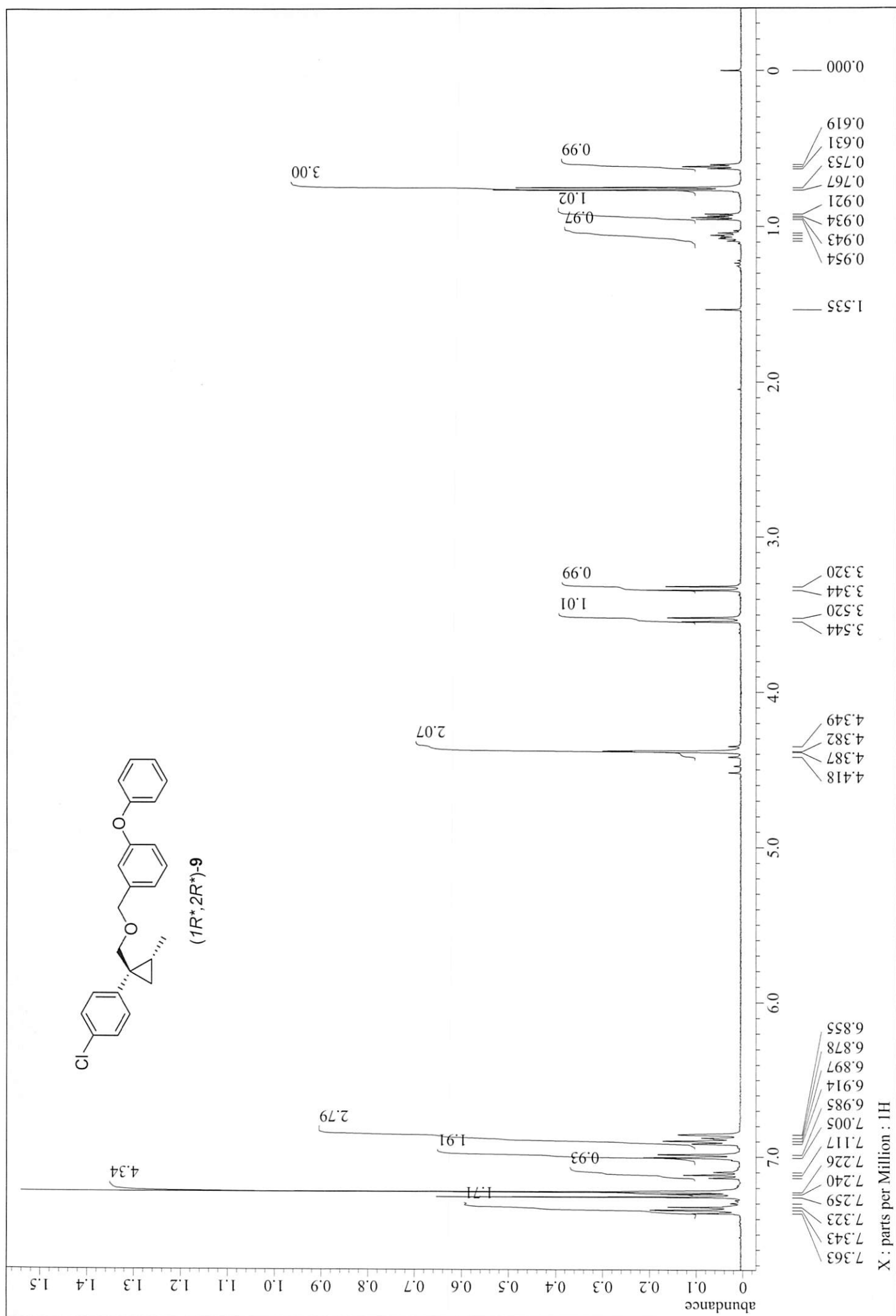

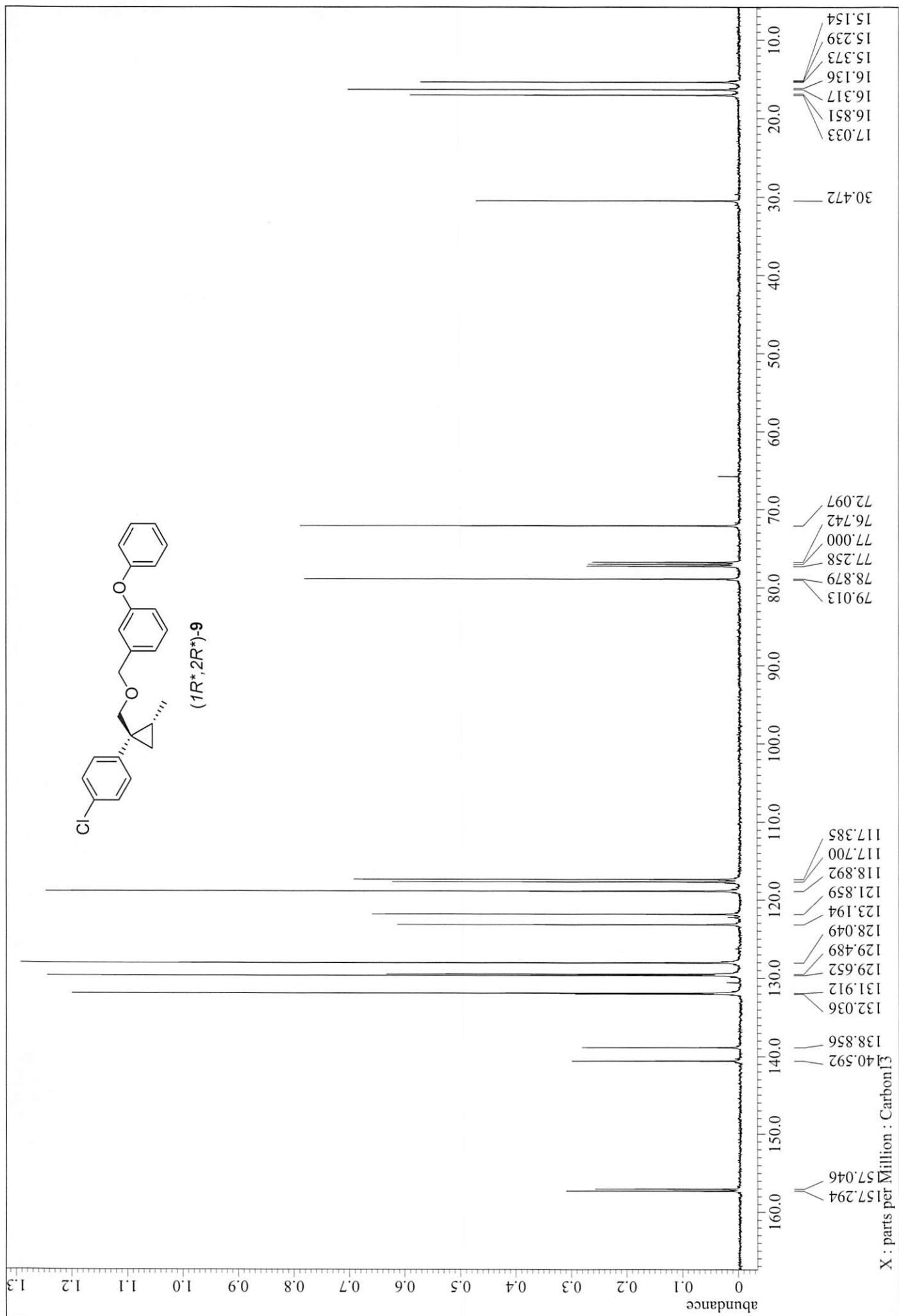

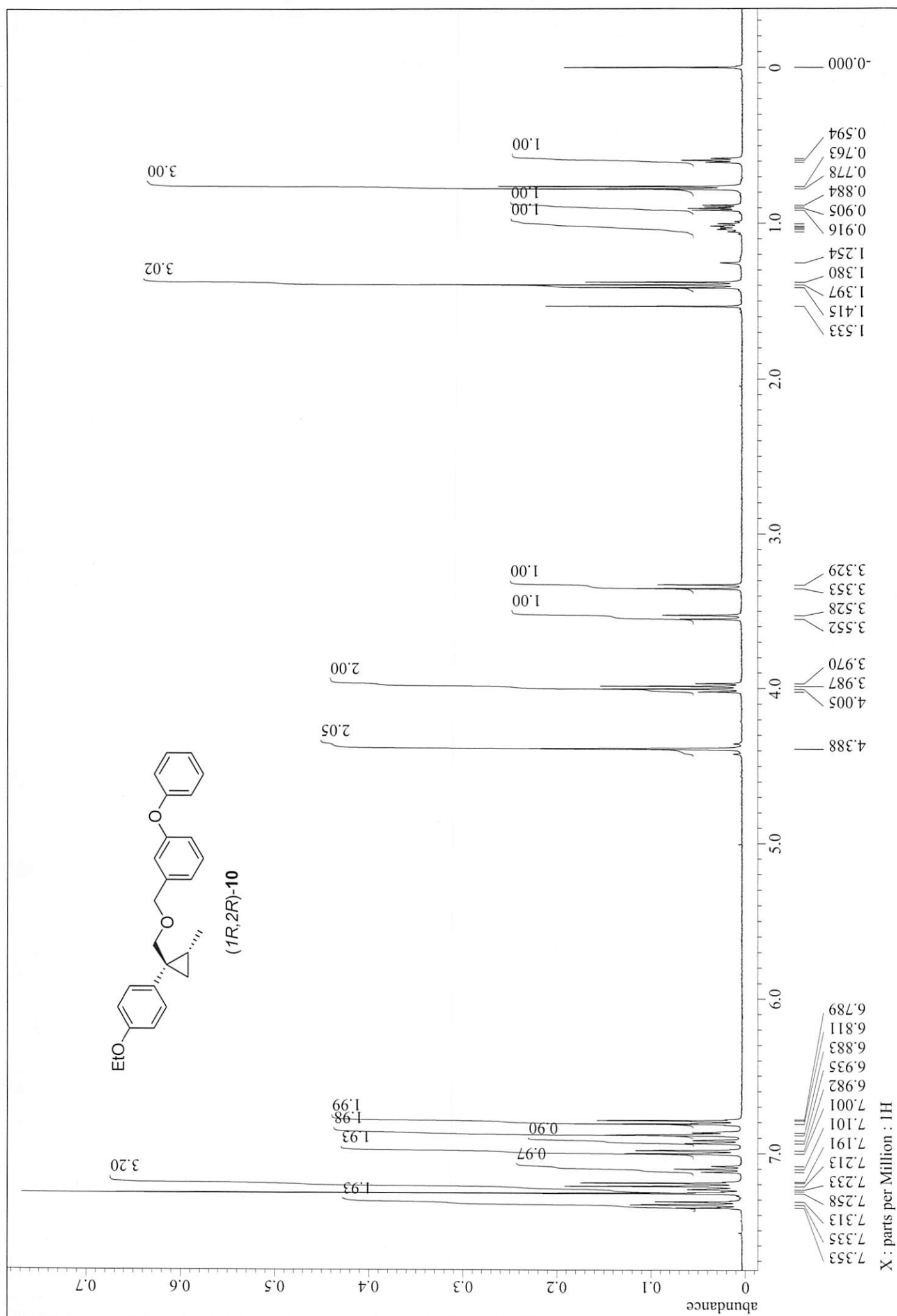

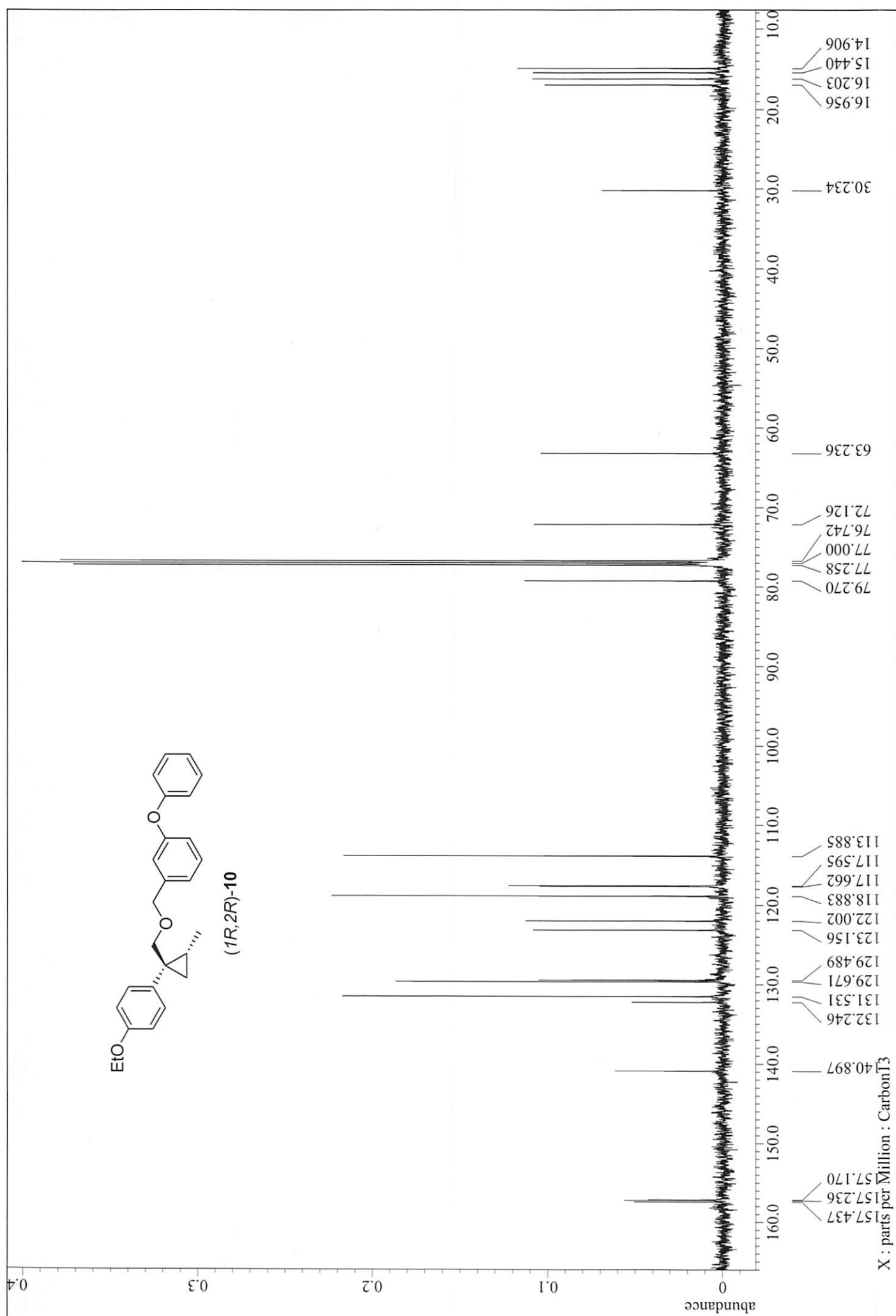

Supplement: Supplementary file 1 [file molecules-24-01023-s001.zip › Molecules-Two asymmetric centers pyrethroid-NMR.pdf]
